# Nanoscale design of an ethylene tetraaniline–bipyridine-based crystalline covalent organic framework for enhanced hydrogen evolution reaction

**DOI:** 10.1039/d6ra06915k

**Published:** 2026-10-28

**Authors:** Satadal Sahoo, Ishita Mukherjee, Nitesh Kumar, Sanhita Maity, A. D. Dinga Wonanke, Matthew A. Addicoat, Anirban Pradhan

**Affiliations:** a Department of Chemistry, Birla Institute of Technology (BIT) – Mesra Ranchi Jharkhand 835215 India anirbanpradhan@bitmesra.ac.in +91 7003322012; b Department of Applied Sciences, Amity University Ranchi Jharkhand India; c School of Science and Technology, Nottingham Trent University Clifton Lane Nottingham NG11 8NS UK

## Abstract

In this work, a two-dimensional covalent organic framework (ETTA–Bpy-COF) with an **sql** topology was synthesized *via* Schiff-base condensation of 4,4′,4′′,4‴-(ethene-1,1,2,2-tetrayl)tetraaniline (ETTA) and [3,3′-bipyridine]-6,6′-dicarbaldehyde (Bpy) under solvothermal conditions, followed by post-synthetic Ni incorporation. The layered porous architecture of the COF facilitates charge transport and provides accessible catalytic sites for the hydrogen evolution reaction (HER). In 0.5 M H_2_SO_4_, the pristine ETTA–Bpy-COF exhibits an overpotential of 247 mV at 10 mA cm^−2^, a charge-transfer resistance of 70.3 Ω and a Tafel slope of 88 mV dec^−1^. Upon Ni incorporation (0.647 wt%), the overpotential decreases to 214 mV, accompanied by a reduced charge-transfer resistance (48.4 Ω) and a lower Tafel slope (61 mV dec^−1^), indicating enhanced HER kinetics. Density functional theory (DFT) calculations reveal favourable electronic modulation after Ni incorporation, consistent with the experimentally observed improvement in catalytic activity. The catalyst also demonstrates good durability after 1000 cycles and maintains stable current density for 15 h under chronoamperometric conditions. These results highlight the effectiveness of Ni incorporation in enhancing the HER performance of ETTA–Bpy-COF.

## Abstract 2

Solvothermal synthesis of ETTA and Bpy yielded the ETTA–Bpy-COF, followed by post-synthetic modification with nickel ions to obtain ETTA–Bpy-Ni COF. Both COFs demonstrated effective electrocatalytic HER performance *via* water-splitting.

## Graphical abstract

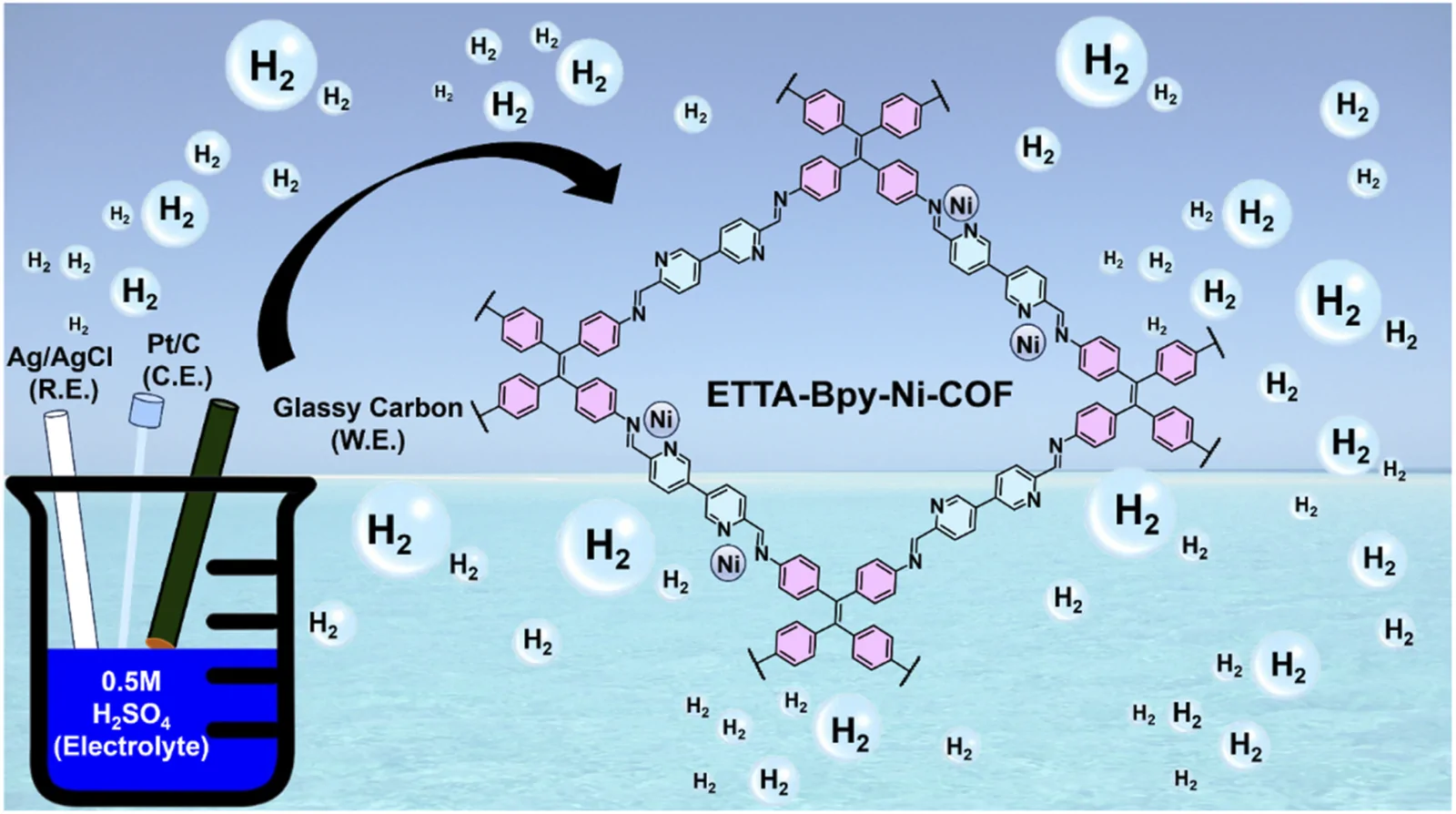

## Introduction

A complex worldwide catastrophe, including severe energy shortages, rapid global warming due to greenhouse gas emissions, and widespread environmental pollution, has been brought on by an overreliance on fossil fuels.^1^ In order to address the energy and environmental issues, finding a practical, clean, and effective energy storage solution has emerged as the most crucial challenge.^2–5^ Among many approaches being investigated, water splitting, which entails the electrochemical breakdown of water into oxygen and hydrogen, stands out as an excellent viable technique for the production of pure hydrogen.^6–8^ It is a matter of interest that hydrogen (H_2_) can be produced in three ways: coal gasification, steam methane reforming and water splitting. Among these three methods, water splitting is the most effective method because the other methods produce a large amount of carbon dioxide. To overcome these obstacles, a sustainable energy conversion system for hydrogen production must be established. Hydrogen is an environmentally friendly renewable energy source.^9^ For this reason, several studies are currently focused on developing commercially feasible catalysts for the production of hydrogen from water.^10^ It is widely accepted that platinum (Pt), iridium (Ir), and ruthenium (Ru) serve as benchmark catalysts for the hydrogen evolution reaction (HER)^11^ but their prohibitive expenses and low relative abundance limit their industrial-scale application for producing hydrogen at a cost-effective rate. In recent decades, some platinum-free transition metal sulphides,^12^ oxides,^13^ phosphides,^14^ selenides,^15^ and carbides^16^ were developed as catalysts for the HER, but the major problem lies with their high Tafel slope and onset overpotential value and low reliability. As a result, this has sparked a critical need to engineer novel robust catalysts, either metal-free or metal-containing, which are significantly effective to produce hydrogen from water electrolytically or photolytically.^17,18^ Recent studies have highlighted the HER activity of some porous architectures including covalent organic frameworks,^19^ metal–organic frameworks,^20^ porous organic polymers,^21^ and covalent organic polymers.^22^ Covalent organic frameworks (COFs)^23–26^ are a special type of crystalline porous material with structural periodicity. COFs are prepared by combining organic building blocks *via* covalent bonding to develop two- or three-dimensional organized structures.^27–30^ However, 2D COFs demonstrate superior performance compared to 3D COFs, which can be attributed to their higher accessible surface area, tunable pore architecture, long-term structural stability, and the inherent π–π stacking interactions between adjacent layers.

These structural features promote efficient charge transport^31,32^ and facilitate enhanced electrocatalytic activity.^33,34^ Recent research provides strong support for the choice of two-dimensional COFs (2D COFs) for the electrocatalytic hydrogen evolution reaction (HER). Song *et al.* reported 2D microporous COFs used as effective supports for cobalt nanoparticles for the electrocatalytic HER. The resulting catalyst exhibited an overpotential of approximately 146 mV at 10 mA cm^−2^ along with a Tafel slope of 84 mV dec^−1^ in alkaline medium, highlighting efficient HER kinetics.^35^ Two crystalline two-dimensional COFs, JLNU-301 and JLNU-302, were synthesised and their electrocatalytic hydrogen evolution capability in both acidic and alkaline conditions was methodically assessed by Fang *et al.* in 2022. To reach a current density of 10 mA cm^−2^ in 0.5 M H_2_SO_4_, JLNU-301 and JLNU-302 needed overpotentials of 189 mV and 151 mV, respectively. For JLNU-301 and JLNU-302, the corresponding Tafel slopes were determined to be 85.41 mV dec^−1^ and 101.56 mV dec^−1^, respectively. They observed improved catalytic activity when evaluated in 1 M KOH. While JLNU-302 displayed a lower overpotential of 91 mV with a Tafel slope of 103.56 mV dec^−1^, JLNU-301 displayed an overpotential of 136 mV with a Tafel slope of 110.58 mV dec^−1^.^36^ Das *et al.* reported a nickel-doped 2D COP (Ni@COP) exhibiting the electrocatalytic HER;^37^ the overpotential and Tafel slope values were 290 mV and 112 mV dec^−1^ in 0.5 M H_2_SO_4_, respectively. The unique properties of COFs have enabled their use in a variety of versatile applications, such as energy storage and conversion,^38–40^ sensing,^41^ gas storage and separation,^42,43^ catalysis,^44^ proton conduction,^45,46^ drug delivery,^47^ and photocatalysis^48^ but their application in the HER is very rare. Owing to their tunable π-conjugated structures, high surface area, and designable pore environments, covalent organic frameworks (COFs) have recently emerged as versatile functional materials, finding applications in optoelectronic devices like OLEDs^49^ and organic solar cells,^50^ flexible electronics,^51^ macrocycles,^52^ biosensing platforms,^53^ catalytic processes like methane dry reforming,^54^ and emerging energy technologies like osmotic power harvesting.^55^ The HER activity of an electrocatalyst primarily depends on two factors: the first is a low overpotential value, and the second is the kinetics of hydrogen production.^56,57^ Porous materials that enable to drive the HER activity can be classified into two categories: metal-free frameworks and metal-coordinated architectures. Over the past few years, our group has reported some metal-free HER active electrocatalysts.^19^ Our research group has recently published a review article related to the advancements of the HER^58^ and is dedicatedly working in this field to synthesize COFs that exhibit superior HER activity. The main focus of this work is to prepare, from two unique types of moieties, a nanoscale 2D COF that demonstrates stability and catalytic activity in the electrochemical HER and potential for enhanced catalytic activity *via* post-synthetic modification by doping with a metal. Herein, we developed a Schiff base-based covalent organic framework through the condensation of 4,4′,4′′,4‴-(ethene-1,1,2,2-tetrayl)tetraaniline (ETTA) and [3,3′-bipyridine]-6,6′-dicarbaldehyde (Bpy) that exhibits the electrocatalytic HER activity. The resultant material was used to create an Ni-COF electrocatalyst to enhance the electrocatalytic activity in an acidic medium.

## Experimental section

### Materials

The reagents were obtained from a commercial source and utilized without any further purification. 4,4′,4′′,4‴-(Ethene-1,1,2,2-tetrayl)tetraaniline (ETTA), [3,3′-bipyridine]-6,6′-dicarbaldehyde (Bpy) and nickel nitrate hexahydrate were purchased from BLD Pharma, 3ASenrise and Spectrochem, respectively. The solvents methanol, *n*-butanol, and acetic acid were purchased from Qualigens, Thermo Fisher Scientific Pvt. Ltd, and *o*-dichlorobenzene was purchased from Spectrochem, India.

### Instrumentation

The Fourier transform infrared spectroscopy (FTIR) data for the ETTA–Bpy-COF and its Ni-analog were recorded using a PerkinElmer Frontier FTIR/FIR spectrometer. Using a JEOL ECX 400, the solid-state cross-polarization magic-angle spinning carbon-13 nuclear magnetic resonance (^13^C CP-MAS NMR) spectra were captured. X-ray photoelectron spectroscopy (XPS) analysis was performed using a Thermo Fisher Scientific Instrument (UK) with a 6 mA beam current and a 12 kV accelerating voltage to determine the carbon and nitrogen concentrations of the samples. The Rigaku (Japan) SmartLab 9 kW instrument (with 2*θ* = 3°–120°) was used to obtain the powder X-ray diffraction (PXRD) patterns. At 77 K, an LX Nova Touch LXI analyzer (Quantachrome Instruments, USA) was used to measure the N_2_ adsorption/desorption isotherms of the synthesized samples. The samples were subjected to 24 h of degassing at 120 °C before measurements. The pore size distribution (PSD) was calculated using the BJH method. A Carl Zeiss Sigma 300 field-emission scanning electron microscope with a magnification of 25× to 1200k× was used in the morphological study of the synthesized compounds. A JEOL JEM F200 with a resolution of 0.19 nm and a voltage of 20–200 kV was used to capture high-resolution transmission electron microscopy (HRTEM) images. Thermogravimetric analysis (TGA) was conducted in the presence of N_2_ using a Shimadzu Corporation DTG-60 instrument over a temperature range from ambient temperature to 1150 °C. To determine the weight percentage of the metal present in the synthesized COF, ICP-OES was conducted using a PerkinElmer instrument. Each electrochemical reaction was carried out in a 0.5 M H_2_SO_4_ electrolyte solution at 27 °C using an Autolab 302N electrochemical analyser potentiostat–galvanostat with a three-electrode configuration. For the linear sweep voltammetry (LSV) investigation, a scan rate of 10 mV s^−1^ was maintained. The catalyst's stability was tested for 1000 cycles at a scan rate of 100 mV s^−1^ between 0.2 V and −0.5 V (*versus* RHE). The impedance measurement's frequency range is 100 kHz to 10 mHz.

#### Synthesis of ETTA–Bpy-COF

4,4′,4′′,4‴-(Ethene-1,1,2,2-tetrayl)tetraaniline (ETTA) (30 mg, 0.0764 mmol) and [3,3′-bipyridine]-6,6′-dicarbaldehyde (Bpy) (32 mg, 0.1508 mmol) were placed in a Schlenk tube. A mixture of *o*-dichlorobenzene and *n*-butanol (4 : 1, v/v) was added, followed by 0.1 mL of 6 M acetic acid. The reaction mixture was sonicated for 30 min to ensure proper mixing. The tube was degassed by three freeze–pump–thaw cycles, sealed under vacuum, and heated at 120 °C for 72 h under static conditions. After cooling to room temperature, an orange precipitate was obtained. The resulting solid was collected by filtration and thoroughly washed with water until the filtrate became colorless.

It was subsequently washed with acetone, followed by tetrahydrofuran (THF) to remove residual impurities. The product was then dried overnight to obtain ETTA–Bpy-COF as an orange-colored powder.

#### Synthesis of ETTA–Bpy-Ni COF

For the synthesis of Ni-doped COF, ETTA–Bpy-COF (30 mg) and Ni (NO_3_)_2_·6H_2_O (30 mg) were placed in a round-bottom flask with methanol (13 mL). The mixture was stirred under a nitrogen atmosphere at room temperature for 12 h. After stirring, the precipitate was collected by filtration and washed several times with methanol to remove any unreacted nickel salt. The resulting material, ETTA–Bpy-Ni COF, was dried at room temperature and was used for further studies.

#### Catalyst preparation

In order to create the catalyst ink, 20 µL of 0.5 weight percent Nafion binder and 1 mg of the catalyst were dissolved in 1 mL of a solvent mixture (IPA : H_2_O = 1 : 1, v/v). Prior to catalyst deposition, the GC electrode was thoroughly cleaned by rinsing with distilled water, followed by polishing with 0.05 µm alumina slurry to obtain a smooth and clean surface. Then, one drop (10 µL) of the prepared catalyst was dropped onto the GC electrode having a diameter of 3 mm, which was then left to air-dry overnight.

## Results and discussion

As shown in Scheme 1, the square lattice-shaped ETTA–Bpy-COF was synthesized using 4,4′,4′′,4‴-(ethene-1,1,2,2-tetrayl)tetraaniline (ETTA) having *C*_2_ symmetry and [3,3′-bipyridine]-6,6′-dicarbaldehyde (Bpy) having *C*_2_ symmetry as precursors. ETTA–Bpy-COF was synthesised by Schiff base condensation using a solvothermal method in the presence of BuOH (0.6 mL) and *o*-DCB (2.4 mL) as a solvent mixture and 6 M acetic acid (0.1 mL) as a catalyst for 72 hours. The linkage and functionality of the synthesized ETTA–Bpy-COF were characterized by FTIR spectroscopy and ^13^C-CPMAS NMR. The crystallinity was confirmed by PXRD. Elemental composition and thermal stability were confirmed by XPS and TGA, respectively. The BET surface area was determined from the N_2_ adsorption–desorption isotherms. The morphology of the synthesised COF was analysed using SEM and HRTEM. The condensation reaction between the two building units was verified by FTIR spectroscopy (Fig. 1A). In the spectra of the individual monomers, a distinct absorption band at 1707 cm^−1^ corresponding to the stretching vibration of the aldehyde (C=O) group and a broad band at 3342 cm^−1^ attributed to the N–H stretching vibration of the amine group were observed. In contrast, these characteristic peaks were absent in the spectrum of the resulting material. The disappearance of these bands confirms the consumption of the aldehyde and amine functionalities, thereby evidencing the successful formation of the condensed framework. A new peak was observed at 1610 cm^−1^ for the synthesised COF, which indicates the presence of the imine (–C=N) linkage.^59^ Additionally, another peak was observed at 1504 cm^−1^, signifying the C=C stretching frequency. After post-synthetic modification, the two characteristic peaks (–C=N– 1610 cm^−1^, –C=C– 1504 cm^−1^) in the same frequency region were observed for the Ni-doped analog; *i.e.*, ETTA–Bpy-Ni COF remained the same^60^ (Fig. 1A). This result confirms the incorporation of Ni into the structural backbone. Furthermore, the presence of the imine group in ETTA–Bpy-COF was proven by ^13^C-CPMAS NMR, which shows a peak at 158 ppm (ref. 61) (Fig. 1B). The porosity of ETTA–Bpy-COF and ETTA–Bpy-Ni COF was verified by nitrogen adsorption–desorption measurements performed at 77 K. The ETTA–Bpy-COF and ETTA–Bpy-Ni COF showed a type IV adsorption isotherm and a hysteresis loop at high relative pressure, suggesting a mesoporous framework.^62^ The BET surface area of ETTA–Bpy-COF (Fig. 1C) and ETTA–Bpy-Ni COF (Fig. 1C) were 108 m^2^ g^−1^ and 67 m^2^ g^−1^, respectively. Using the Barrett–Joyner–Halenda (BJH) method, the pore size distribution of ETTA–Bpy-COF and ETTA–Bpy-Ni COF was analysed, revealing an average pore diameter of 4.5 nm and 4.3 nm, further confirming the mesoporous nature of the synthesized frameworks (Fig. 1C). The adsorption of Ni ions into the pores resulted in a decreased surface area and pore diameter. The surface chemical composition and the corresponding chemical states of the synthesized ETTA–Bpy-COF were investigated using XPS. The XPS survey spectrum (Fig. S10) shows two prominent peaks centered at approximately 284 eV and 399 eV, which are attributed to the C 1s and N 1s core-level signals, respectively. Furthermore, deconvolution of the two broad spectra of C 1s and N 1s provided accurate information about the chemical state of the corresponding elements. The deconvoluted spectrum of C 1s suggests three peaks at the binding energies of 283.9, 285 and 286 eV, which were assigned as C=C, C=N, and C–N bonds, respectively^63^ (Fig. S10). According to the COF structure, there were two types of nitrogen, and the deconvoluted spectrum of N 1s suggests two peaks at the binding energies of 398.6 and 399.9 eV, which were assigned as imine and pyridine nitrogens, respectively^63^ (Fig. S10). The incorporation of Ni into the ETTA–Bpy-COF framework was confirmed by XPS. In the high-resolution Ni 2p spectrum, two main peaks located at approximately 856.0 and 873.4 eV are assigned to Ni 2p_3/2_ and Ni 2p_1/2_, respectively, along with their associated satellite features (Fig. 1F). These characteristics are consistent with the presence of Ni in the +2 oxidation state. Changes in the N 1s spectrum further provide insight into the coordination environment. In comparison to the pristine COF, the binding energies of both the imine (–C=N–) nitrogen and bipyridine nitrogen shift toward higher values after Ni incorporation, appearing at around 399 eV and 401 eV, respectively (Fig. 1E). This shift indicates a decrease in electron density around the nitrogen atoms, suggesting that Ni^2+^ interacts with both nitrogen sites. These observations support the formation of a coordinated Ni–N environment within the framework.^64^ After the incorporation of Ni, the deconvoluted spectrum of C 1s appeared similar to that of the pristine one (Fig. 1D). The crystallinity of ETTA–Bpy-COF was confirmed by PXRD (Fig. 2A), structural simulation, and Pawley refinement factor. According to PXRD, the 2*θ* value arises at 3.440°, which corresponds to the (100) lattice plane, confirming the crystallinity of the compound, and a hump observed at the 2*θ* value of 23.510° is attributed to the π–π stacking between two adjacent layers of ETTA–Bpy-COF.^64^ In the case of ETTA–Bpy-Ni COF, a hump was observed at 25.20°, justifying the presence of Ni incorporated into the COF structure (Fig. S1).^65^ After the addition of Ni to the pristine COF, we observed a shift of our first peak from 2*θ* = 3.440° to 3.822°. Thus, a larger diffraction angle might be attributed to a smaller pore size after post-metalation inside the pores of ETTA–Bpy-COF.^66^ An increase in the intensity of the Ni-analogue was observed when compared to pristine COF. According to structural simulation, the ETTA–Bpy-COF structure perfectly matched slipped *XY* stacking (Fig. 2E), achieving square-shaped platforms and a *P*1 space group possessing unit cell parameters *a* = 23.547558, *b* = 23.792091, *c* = 9.614005 and *α* = 88.92778, *β* = 91.530137, *γ* = 68.961173. Furthermore, the agreement between the simulated and experimental structures was confirmed by Pawley refinement, yielding profile residual (*R*_p_) and weighted profile residual (*R*_wp_) values of 2.88% and 5.12%, respectively, indicating a good fit between the proposed model and the experimental data (Fig. 2D). The chemical stability of ETTA–Bpy-COF was investigated in both alkaline 1 M NaOH and acidic 1 M H_2_SO_4_ media for two days (Fig. S5). The thermal stability of the synthesised COFs was determined by TGA and according to this analysis, ETTA–Bpy-COF was observed to be stable up to 400 °C (Fig. S3). The surface morphology of the synthesised ETTA–Bpy-COF was studied by SEM (Fig. 3A and B) and HRTEM (Fig. 3C). The ETTA–Bpy-COF exhibits a distinct needle-like morphology as revealed by SEM. The layered sheet-like morphology was established from the HR-TEM study. In addition, the crystallinity of the COF was proved by HR-TEM after observing fringes at 5 nm (Fig. 3D). After applying the fast Fourier transform (FFT) to the selected region of the COF (Fig. 3E), it was observed that the orientation of the square pore (002) with a *d*-spacing value of 0.48 nm (Fig. 3F) is well-matched with the theoretically refined structure (*d* = 0.44 nm) (Table S2) and the interlayer distance of 0.42 nm (Fig. 2F). This value highlights the presence of strong π–π stacking in between two adjacent layers. The interlayer distance and π–π stacking strength are inversely proportional to each other; the larger the interlayer distance, the lower the π–π stacking strength and *vice versa*. SEM analysis revealed the morphology of ETTA–Bpy-Ni COF, with a similar needle-like shape to that of the pristine COF (Fig. S14). The elemental mapping images revealed the structural homogeneity of C, N, and Ni ions (Fig. S9). Energy-dispersive X-ray (EDX) spectra confirmed the presence of C, N and Ni in ETTA–Bpy-Ni COF (Fig. S12). To calculate the atomic weight percentage, we performed ICP-OES analysis and the amount of Ni present in the COF was found to be 0.647% (ref. 67 and 68) (Table S1). The improved catalytic performance can be attributed to the strong interaction between the Ni centers and the organic framework. This interaction promotes efficient electron transfer across the structure, leading to enhanced electrical conductivity and increased electron density. The superior activity observed for the HER in acidic conditions further suggests that the electron density around the Ni active sites is significantly elevated, which facilitates faster reaction kinetics.

**Scheme 1:**
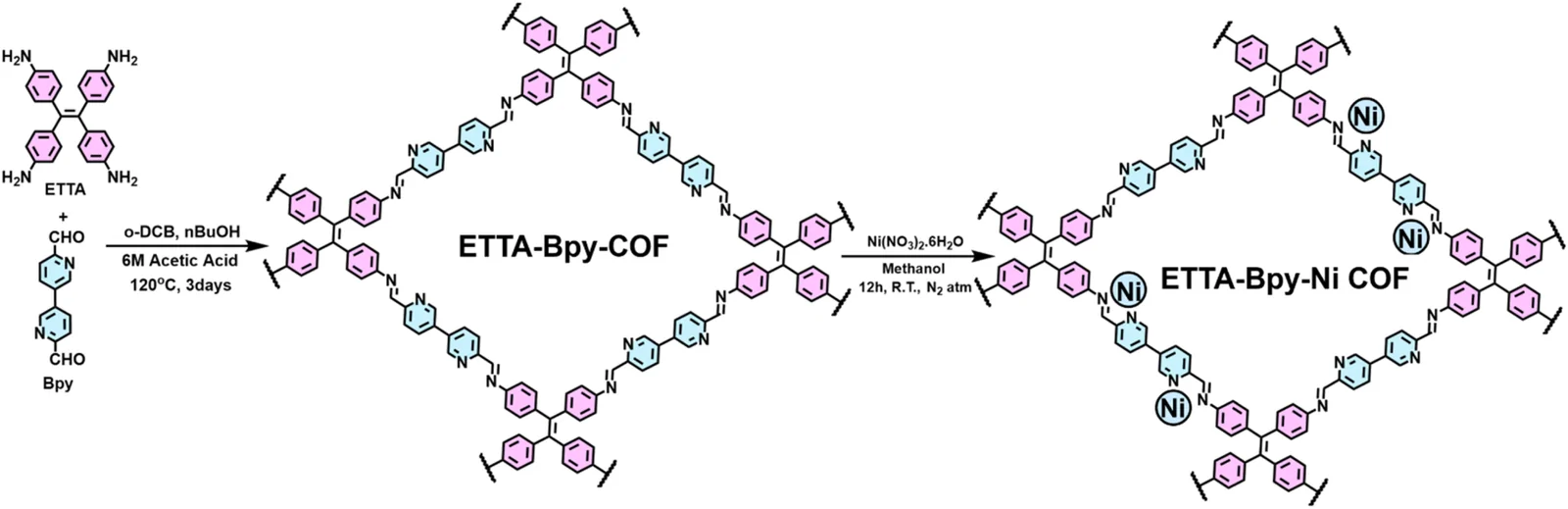
Synthetic scheme of ETTA–Bpy-COF and ETTA–Bpy-Ni COF.

**Fig. 1:**
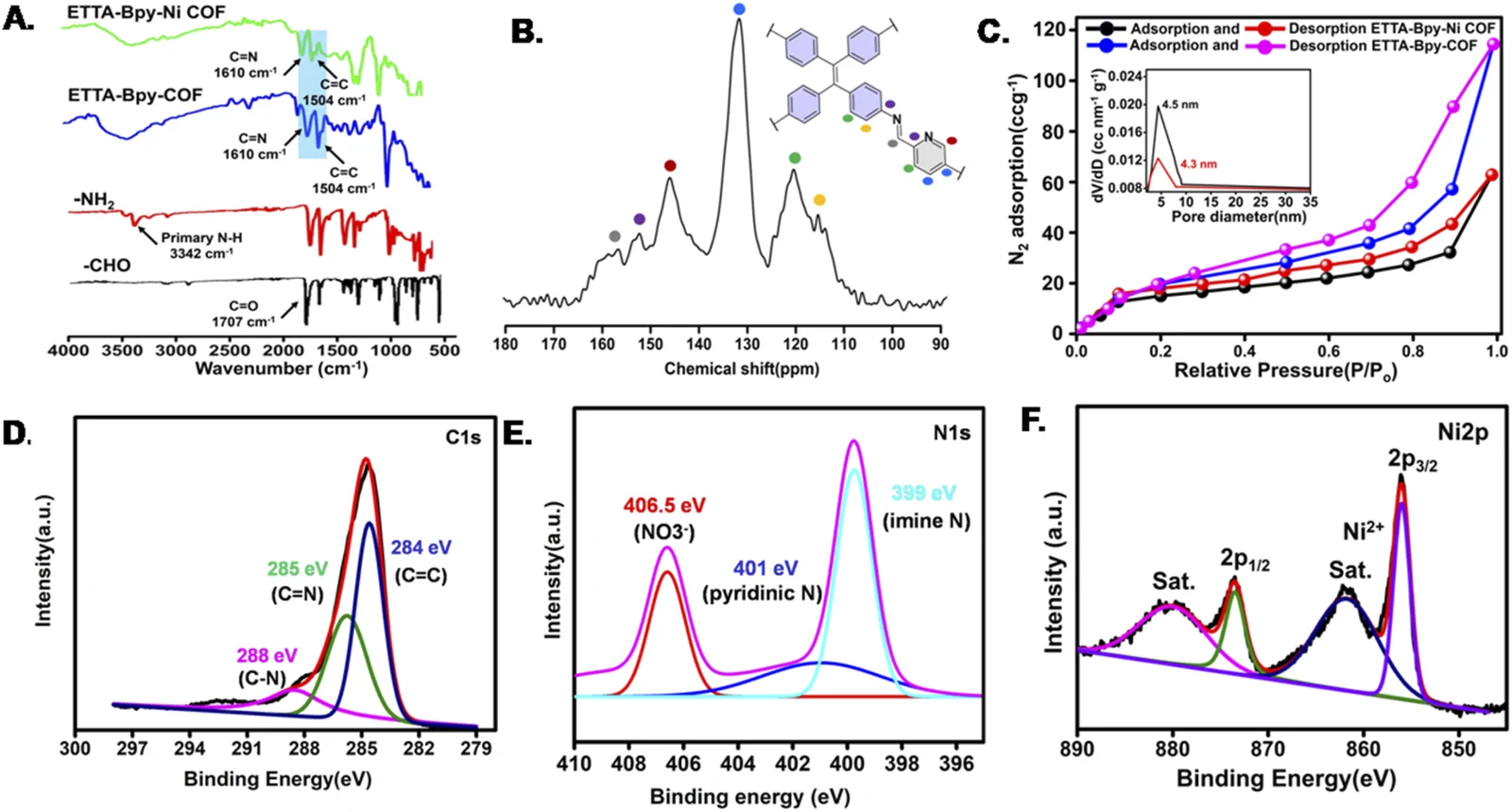
(A) FTIR spectra of ETTA–Bpy-COF and ETTA–Bpy-Ni COF. (B) ^13^C solid-state CP-MAS NMR spectra of ETTA–Bpy-COF. (C) N_2_ adsorption/desorption isotherms at 77 K of ETTA–Bpy-COF and ETTA–Bpy-Ni COF (inset: pore size distribution curves). (D) Deconvoluted C 1s XPS spectra of ETTA–Bpy-Ni COF. (E & F) N 1s and Ni 2p XPS spectra of ETTA–Bpy-Ni COF.

**Fig. 2:**
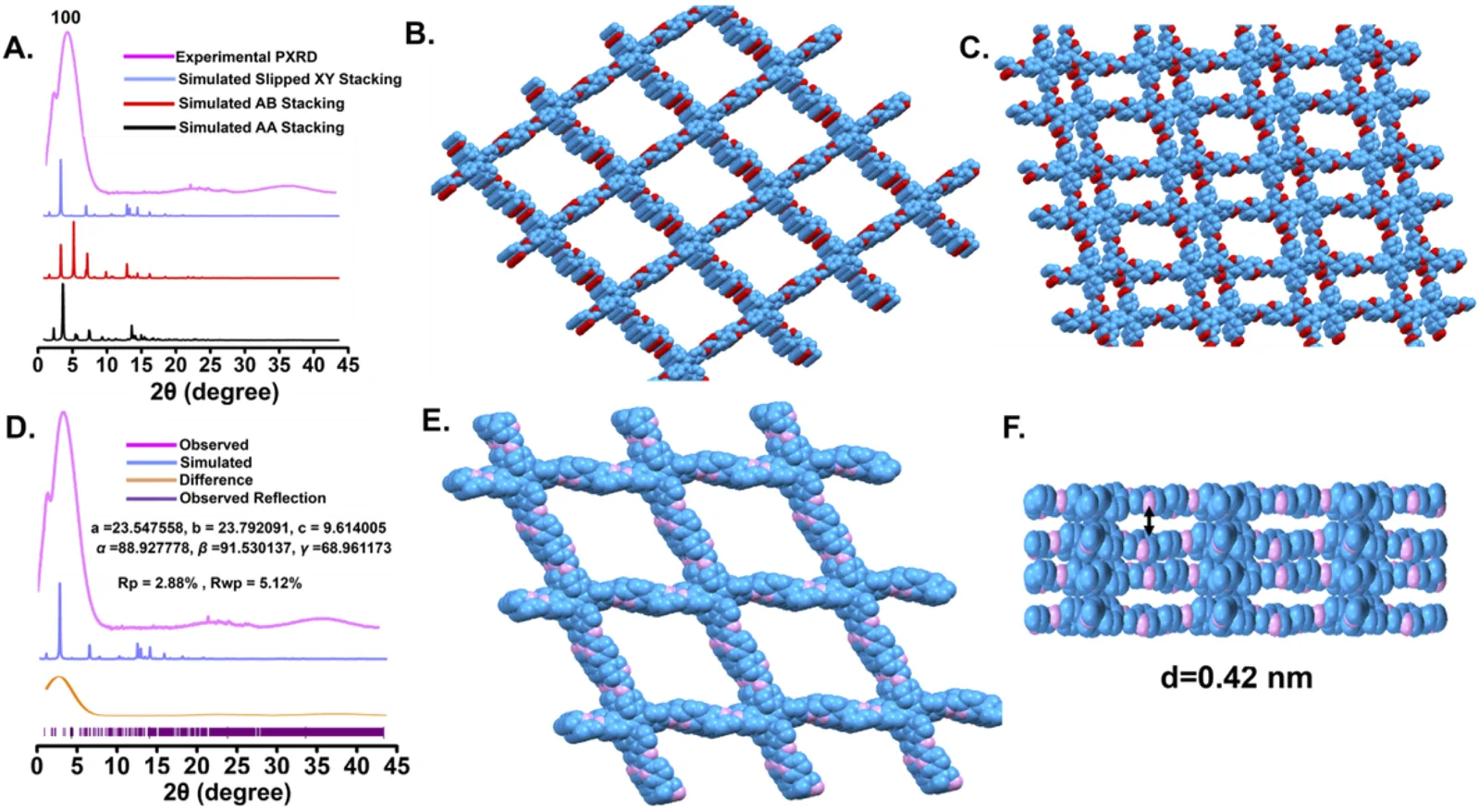
(A) Experimental PXRD patterns. (B) Theoretical AA stacking. (C) Theoretical AB stacking. (D) Pawley refinement of PXRD patterns. (E and F) Front and side views of the simulated slipped *XY* stacking.

**Fig. 3:**
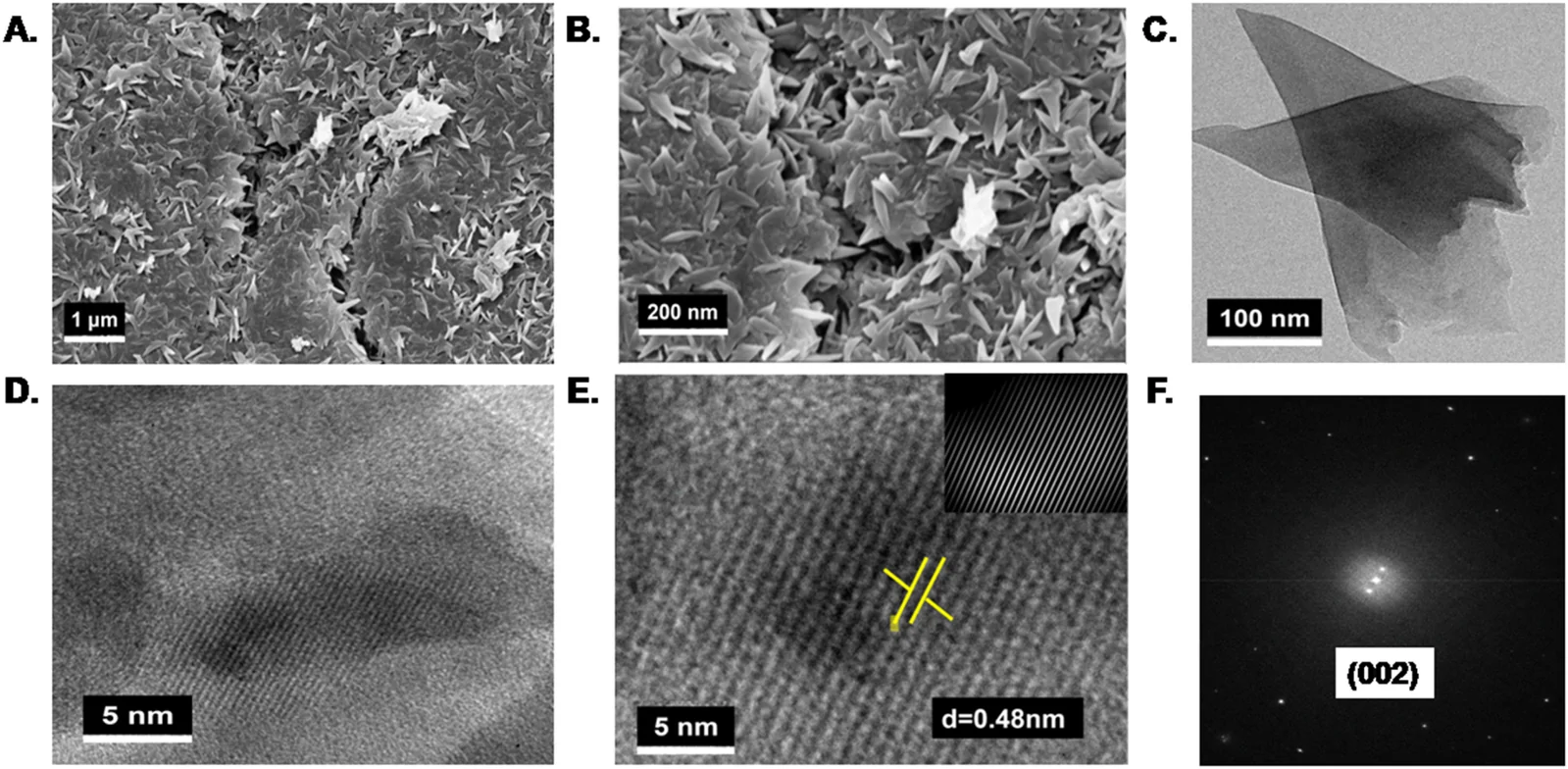
(A and B) SEM images of ETTA–Bpy-COF, (C and D) HRTEM images of ETTA–Bpy-COF, (E) FFT pattern of ETTA–Bpy-COF, and (F) SAED pattern of ETTA–Bpy-COF highlighting the (002) plane.

### Electrochemical HER activity

LSV measurements were conducted in a three-electrode system using the as-synthesised ETTA–Bpy-COF and ETTA–Bpy-Ni COF drop-casted on a glassy carbon (GC) electrode as the working electrode, Ag/AgCl as the reference electrode, and Pt wire as the counter electrode in 0.5 M H_2_SO_4_ electrolyte at 27 °C. The onset of the HER activity at the surface of the catalyst-modified electrode is evidenced by the emergence of a cathodic (negative) current. The bare glassy carbon (GC) electrode exhibited a very low cathodic current, indicating negligible hydrogen evolution reaction (HER) activity. On the other hand, the catalyst-coated GC electrode displayed a noticeable rise in cathodic current, indicating an increase in the electrocatalytic HER activity of the ETTA–Bpy-COF and ETTA–Bpy-Ni COF. The overpotential value is an essential metric for assessing the HER activity since it indicates the degree of polarization associated with the faradaic current during the HER. A more effective catalyst that requires less applied cathodic bias to induce hydrogen generation is indicated by a lower overpotential. The overpotential needed to reach a current density of 10 mA cm^−2^ is typically used as a benchmark for evaluating catalyst efficiency towards the HER. The synthesized electrocatalysts ETTA–Bpy-COF and ETTA–Bpy-Ni COF achieved a current density of 10 mA cm^−2^ at overpotentials of 247 mV and 214 mV, respectively. These values are 189 mV and 156 mV more negative than that of the benchmark Pt/C catalyst (58 mV) under identical conditions (Fig. 4A). These values suggest that our synthesised COFs exhibit promising electrocatalytic HER activity compared to the previously reported electrocatalysts (Table S6A), and after the incorporation of Ni, the electrocatalytic HER activity is enhanced. COFs may accommodate a variety of metal-doped nanostructures within their porous structure due to their enormous surface area, low density, and two-dimensional framework structure. They provide a large specific surface area for Ni ion inclusion, preventing Ni ion agglomeration. This distribution maximizes the use of active sites and encourages effective mass transport. Additionally, they serve as a scaffold for Ni ions, enhancing the material's toughness, stability, and heterogeneity. The presence of several functional groups within the porous 2D COF, such as imine nitrogen and bipyridine nitrogen, can interact with the H_2_O molecules *via* H-bonding. As a result, better contact between the catalyst and electrolyte is possible. Additionally, proton conductivity is increased, which makes it easier for protons to flow quickly toward the active sites. The overall electrocatalytic activity is improved as a result of the increased conductivity, which significantly accelerates the HER process. Next, we checked the electrocatalytic HER activity of ETTA–Bpy in basic medium (Fig. S9), but the current density did not reach 10 mA cm^−2^. The HER activity of the COFs shows strong dependence on the electrolyte pH. In acidic medium, the reaction proceeds *via* the direct reduction of protons (H^+^/H_3_O^+^), which are readily available in high concentrations, leading to fast reaction kinetics. In contrast, in alkaline medium, water (H_2_O) acts as the proton source, and an additional water dissociation step (H_2_O + e^−^ → H* + OH^−^) is required to generate adsorbed hydrogen intermediates. This step is kinetically sluggish and is generally considered the rate-determining step, resulting in significantly reduced HER activity. Furthermore, in the present ETTA–Bpy-based COF system, the catalytic sites are primarily associated with nitrogen functionalities and coordinated Ni centers (Ni–N_2_). Under alkaline conditions, the surface may undergo modification due to adsorption of OH^−^ species, which can block active sites and hinder proton adsorption. Additionally, the protonation of nitrogen sites facilitates hydrogen adsorption in acidic media and is suppressed in alkaline conditions, further reducing catalytic efficiency. It is well reported in the literature that the HER kinetics in alkaline media are typically 2–3 orders of magnitude slower than in acidic media due to the additional water dissociation step and lower proton availability. These factors collectively explain the observed lower HER activity of the COF in basic medium.^69^ This means that the material exhibited substantial HER activity only in acidic medium. The value obtained from the Tafel slope indicates the kinetics and mechanism of the HER for this specific catalyst. This Tafel slope value is determined from the Tafel equation, which is derived from the Butler–Volmer equation:*η* = *a* + *b* log(*i*/*i*_0_)where *η* = overpotential, *a* = constant, *b* = Tafel slope, *i* = measured current density, *i*_0_ = exchange current density. Usually, the HER follows three basic steps. Proton adsorption takes place in the electrocatalyst's pores in the first phase. These deposited protons subsequently combine with electrons to create H*, which is referred to as the Volmer step. The second or third step is the next phase in the development of hydrogen. H* combines with a proton and an electron in the second stage, known as the Heyrovsky reaction, to produce molecular hydrogen. Alternatively, two H* might combine to generate hydrogen gas in the third step of the process, known as the Tafel mechanism. Therefore, the Volmer–Heyrovsky and Volmer–Tafel pathways are the two probable pathways by which hydrogen evolution might happen. The synthesized ETTA–Bpy-COF's Tafel slope was found to be 88 mV dec^−1^ and after the addition of Ni, *i.e.*, ETTA–Bpy-Ni COF, the value was 61 mV dec^−1^ (Fig. 4B). In contrast, the benchmark platinum electrode exhibited a significantly lower Tafel slope of 30 mV dec^−1^, indicating faster reaction kinetics. This observation suggests that the HER on the surfaces of ETTA–Bpy-COF and ETTA–Bpy-Ni COF proceeds through a Volmer–Heyrovsky mechanism, with the Volmer step acting as the rate-determining step. The nitrogen atoms of the bipyridine moiety and the imine nitrogen group are the two unique active sites for hydrogen evolution in the synthesized COF. Proton adsorption takes place at both nitrogen sites in 0.5 M H_2_SO_4_, creating a positively charged species that is then balanced by sulfate ions (SO_4_^2−^). Additionally, the interaction between adsorbed protons (H^+^) and H* at nearby imine nitrogen sites promotes effective hydrogen evolution and aids in the synthesis of hydrogen. Along with the Tafel slope, the exchange current density is another important parameter for evaluating the electrocatalytic performance of ETTA–Bpy-COF. A higher exchange current density reflects more favorable electrode kinetics and improved intrinsic catalytic activity. The exchange current density of ETTA–Bpy-COF and ETTA–Bpy-Ni COF are 2.1 × 10^−4^ A cm^−2^ and 2.78 × 10^−4^ A cm^−2^, respectively. Additionally, the double-layer capacitance (*C*_dl_) was used to calculate the catalyst's electrochemically active surface area (ECSA). The CV curve used to determine the *C*_dl_ value is shown in Fig. S2. Since the ECSA value and *C*_dl_ are directly correlated, an increase in the *C*_dl_ value will also result in an increase in the ECSA value (Fig. S2) illustrates that the ETTA–Bpy-Ni COF has a larger ECSA value 0.271 cm^2^ than the pristine ETTA–Bpy-COF 0.147 cm^2^. This high ECSA value for the ETTA–Bpy-Ni COF also implies an ease of electron transport, enhancing Ni's catalytic activity when incorporated into the COF structure.

**Fig. 4:**
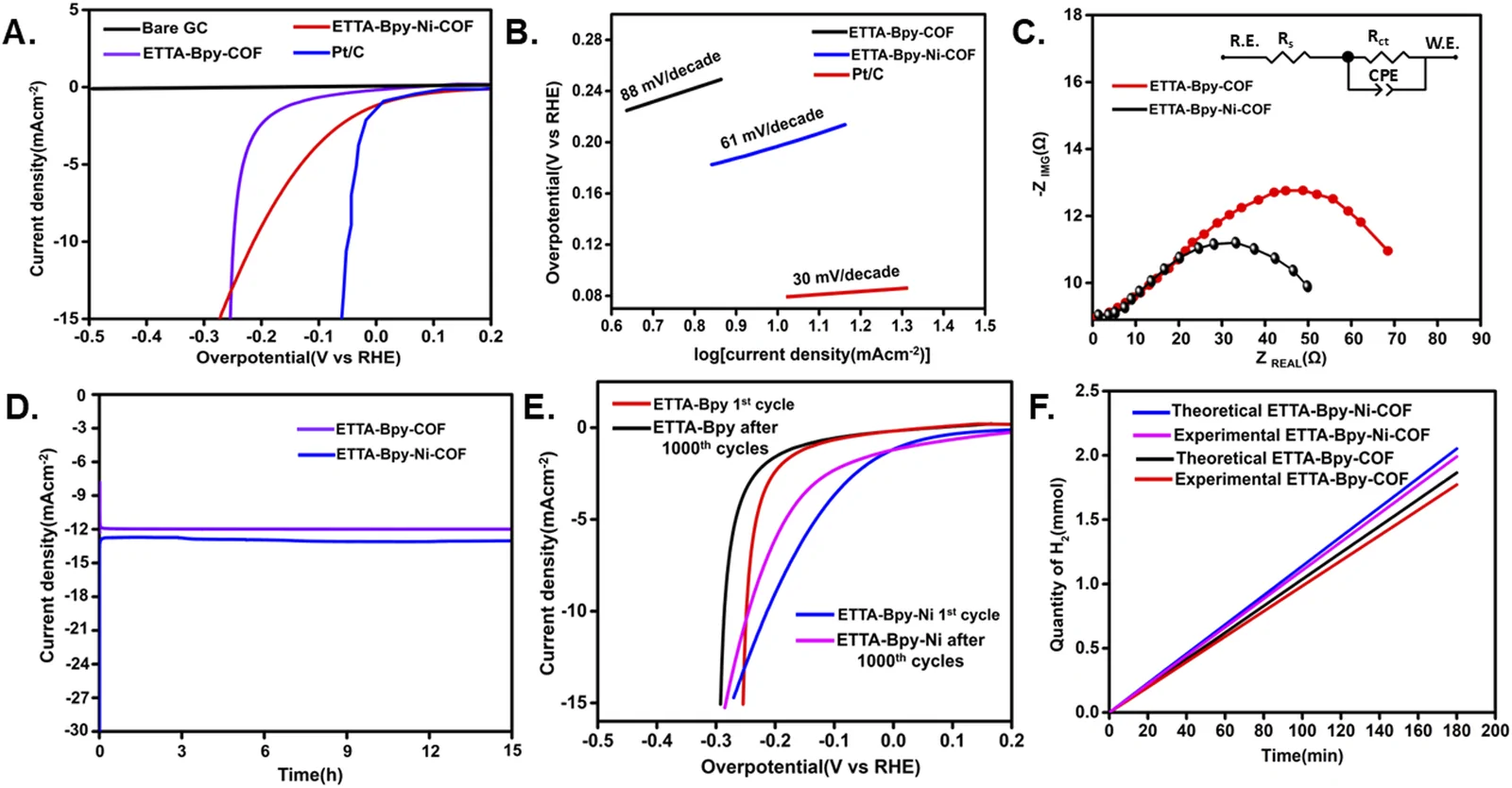
(A) LSV plots of ETTA–Bpy-COF, ETTA–Bpy-Ni COF, Pt/C and bare GC in 0.5 M H_2_SO_4_. (B) Tafel plots of ETTA–Bpy-COF and ETTA–Bpy-Ni COF. (C) Nyquist plots of ETTA–Bpy-COF and ETTA–Bpy-Ni COF. (D) Chronoamperometry plots of ETTA–Bpy-COF and ETTA–Bpy-Ni COF. (E) LSV plots of ETTA–Bpy-COF and ETTA–Bpy-Ni COF in their 1st and 1000th cycles. (F) Faradaic efficiency for H_2_ generation by ETTA–Bpy-COF and ETTA–Bpy-Ni COF.

To further understand the origin of the enhanced electrocatalytic activity, the interfacial charge-transfer properties of the modified electrode were investigated. Fig. 4C presents the Nyquist plot of the ETTA–Bpy-COF; the value of the charge-transfer resistance value can be calculated from it. The charge-transfer resistance (*R*_ct_) corresponds to the diameter of the semicircular arc observed for ETTA–Bpy-COF, which is 70.3 Ω and for ETTA–Bpy-Ni COF, it is 48.4 Ω, which is comparable with the previously reported catalyst (Table S6A). The lower charge transfer resistance value indicates that charge transfer can occur easily between the electrode and electrolyte, which determines the better electrocatalytic performance of the ETTA–Bpy-COF and ETTA–Bpy-Ni COF. The extended conjugated π electronic structure of the ETTA–Bpy electrode is responsible for its high charge-transfer capacity. Fig. 4E presents the long-term potentiometric durability test of the as-synthesized COF to determine the HER activity of the ETTA–Bpy-COF- and ETTA–Bpy-Ni COF-coated GC electrodes after 1000 cycles. Since the catalytic performance of a material is strongly associated with its long-term operational stability, durability studies were conducted for the COFs over 1000 cycles. The results confirmed that the stability of the COF is comparable to previously reported systems, retaining 89% of its initial activity. Moreover, its Ni-incorporated analogue, ETTA–Bpy-Ni COF, exhibited improved stability with 91% retention after the cycling test. The durability test for a long-term period in chronoamperometry of this ETTA–Bpy-COF is determined by the time-dependent current profile (Fig. 4D). From the time-dependent current profile diagram, it was observed that the ETTA–Bpy-COF and its analogue showed a constant current density for 15 hours at −0.3 V *vs.* RHE. Therefore, both long-term chronoamperometry and long-term potentiometry indicate the stability of the synthesised COFs (Fig. 4D and E). Additionally, an electrocatalyst's potential for the effective use of electrical energy for electrocatalysis is implied by its faradaic efficiency. The faradaic efficiency of the ETTA–Bpy-COF was calculated using the following equation:
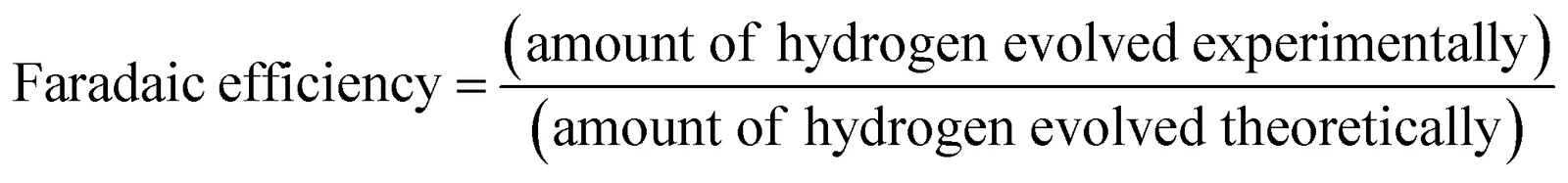


The faradaic efficiency of the ETTA–Bpy-COF and ETTA–Bpy-Ni COF was determined to be 95% and 97%, respectively, which are quite close to the ideal value (Fig. 4F).

### Theoretical calculations

The HER activity of the pristine ETTA–Bpy-COF and Ni-doped ETTA–Bpy-Ni COF was further investigated using density functional theory based on a single-layer cluster model carried out using the ORCA r^2^SCAN-3c level of theory. Multiple hydrogen adsorption configurations were generated by placing H* at the different chemically accessible nitrogen sites and around the Ni centre, as illustrated in Fig. S19 and S20 for the pristine and Ni-doped COF, respectively. The calculated hydrogen adsorption free energies (Δ*G*_H*_) illustrated in Fig. 5 show that the site-dependent catalytic behavior is very significant. In the pristine COF, the adsorption energies span from strongly exergonic to weakly endergonic values (−0.281 to 0.475 eV), indicating heterogeneous proton binding environments across the framework.

**Fig. 5:**
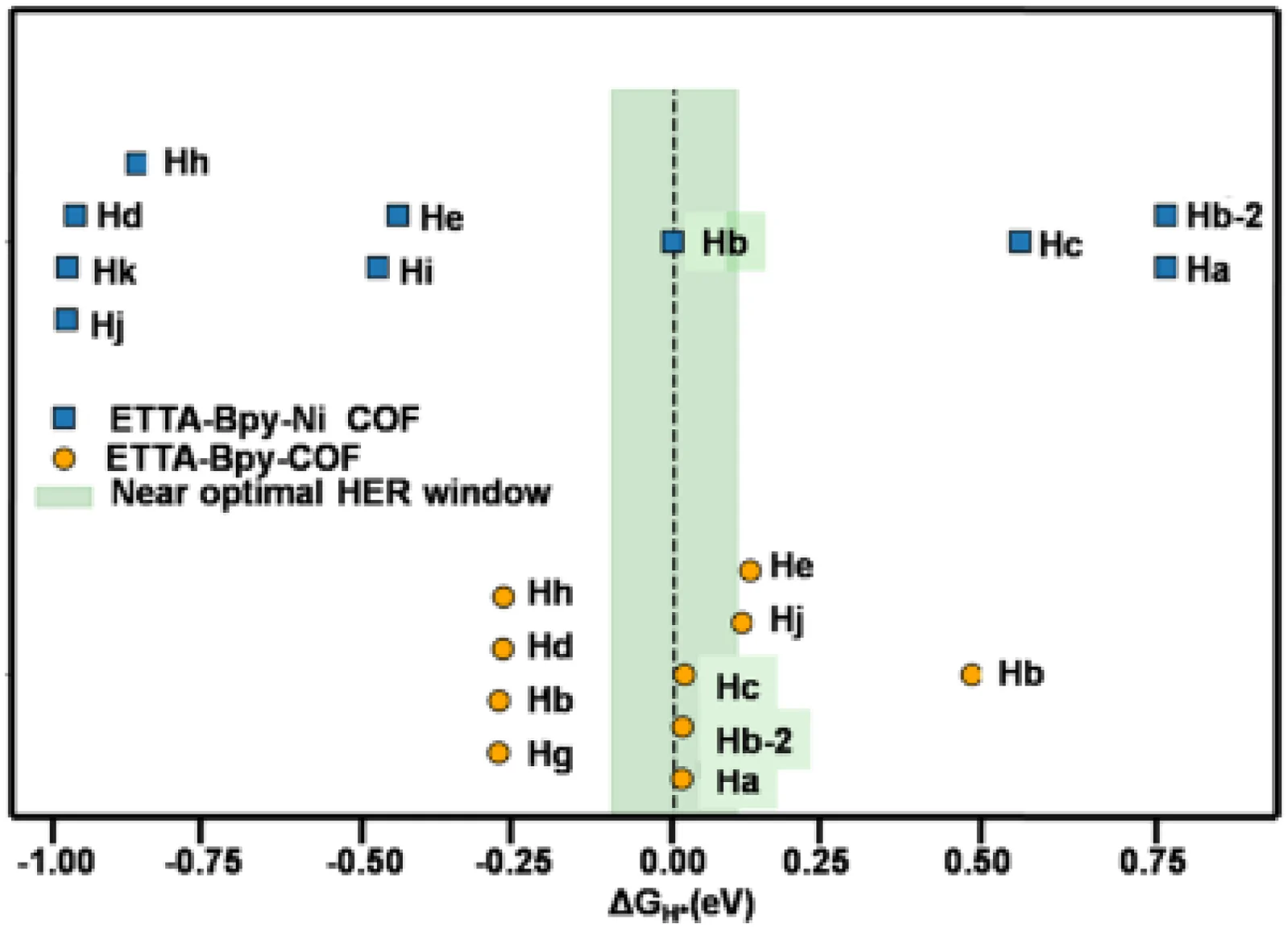
Gibbs free energy of hydrogen adsorption (Δ*G*_H*_) for ETTA–Bpy-COF and ETTA–Bpy-Ni COF at different adsorption configurations.

Among the investigated configurations, Ha, Hb-2 and Hc exhibited adsorption free energies closest to the thermoneutral region (Δ*G*_H*_ ≈ 0) and the value of |Δ*G*_H*_| is 0.013 eV, suggesting comparatively favorable the HER activity according to the Sabatier principle (Table S4). These active configurations are primarily associated with pyridinic nitrogen atoms, indicating that pyridine-based nitrogen sites act as the dominant catalytic centers in the metal-free framework. Upon Ni incorporation, a significant modulation of the hydrogen adsorption energetics was observed. The Ni-doped ETTA–Bpy-Ni COF exhibited a broader distribution of adsorption energies (−0.971 to 0.785 eV), together with the emergence of new metal-assisted adsorption environments.

Most notably, the Hb configuration shows a nearly thermoneutral hydrogen adsorption free energy and the value of |Δ*G*_H*_| is 0.003 eV, indicating an energetically optimal HER adsorption/desorption balance (Table S5). Furthermore, configurations such as He, Hi, Hj, and Hk become substantially stabilized after Ni doping, demonstrating the strong electronic influence of the metal centre on the surrounding conjugated framework. The improved hydrogen adsorption thermodynamics correlate well with the experimentally observed reduction in overpotential and lower Tafel slope for the Ni-doped COF. These results suggest that Ni incorporation electronically activates nearby nitrogen sites while simultaneously introducing additional proton adsorption pathways that are absent in the pristine material. Charge analysis further confirms that hydrogen adsorption is strongly governed by the local electronic environment of the nitrogen sites. In pristine ETTA–Bpy-COF, the H* species carries a positive charge of *ca.* +0.41 to +0.47, while the directly bonded nitrogen atoms become more negatively charged, typically reaching −1.13 to −1.14 for the most active configurations. This indicates a polar N–H interaction in which electron density is localized on the pyridinic nitrogen site, stabilizing the adsorbed proton. The configurations closest to thermoneutral adsorption, particularly Ha, Hb-2 and Hc, involve pronounced charge accumulation on N-2/N-1, consistent with these pyridinic sites acting as the preferred proton-binding centres in the metal-free framework. For the Ni-doped COF, Ni doping redistributes the charge across the coordination environment and adjacent nitrogen atoms. The Ni centre remains positively charged in the host structure, but its charge decreases markedly for Ha and Hb-2, indicating electron density redistribution from the metal node upon H* adsorption. In most Ni-doped configurations, H* remains positively charged (+0.38 to +0.46), while the bonded nitrogen atoms retain strongly negative charges of approximately −1.12 to −1.15, supporting proton stabilization through N-centred adsorption. Notably, the nearly thermoneutral Hb configuration combines a moderately positive H* charge with a strongly negative neighboring nitrogen environment, suggesting an optimal balance between proton binding and desorption. The unusual negative H* charge observed for Hj and Hk results from the Ni–H* bond, which is consistent with overly stabilized adsorption and poorer HER thermodynamics.

## Conclusions

In summary, ETTA–Bpy-COF, a crystalline two-dimensional imine-linked COF, and its Ni-incorporated equivalent were effectively synthesised and investigated as electrocatalysts for the HER in 0.5 M H_2_SO_4_ media. The development of a nanoscale, porous and chemically stable layered framework was verified by structural characterisation. The pristine COF demonstrated good HER activity with an overpotential of 247 mV at 10 mA cm^−2^. However, Ni inclusion greatly improved the performance, reducing the overpotential to 214 mV and the Tafel slope to 61 mV dec^−1^. Enhanced charge transfer and the creation of Ni–N active sites within the framework are the sources of increased activity. Over an extended period of operation, both catalysts also showed great faradaic efficiency and outstanding electrochemical stability. In order to create stable and effective non-noble metal HER electrocatalysts for sustainable hydrogen production, this study emphasises the usefulness of post-synthetic metal integration in 2D COFs.

## Author contributions

All the authors contributed equally.

## Conflicts of interest

The authors declare no conflicts of interest.

## Supplementary Material

RA-OLF-D6RA06915K-s001

## Data Availability

The data supporting this article have been included as part of the supplementary information (SI). Supplementary information is available. See DOI: https://doi.org/10.1039/d6ra06915k.
