# Supplementary material for: Nanoscale design of an ethylene tetraaniline–bipyridine-based crystalline covalent organic framework for enhanced hydrogen evolution reaction
Source: RSC Adv. 2026 Oct 28;16(49):66892–902. doi: 10.1039/d6ra06915k (PMC13715143; doi:10.1039/d6ra06915k)
Supplement: Supplementary file 1 [file RA-016-D6RA06915K-s001.pdf]

Supplementary Information (SI) for RSC Advances.
This journal is © The Royal Society of Chemistry

Supporting Information

Nanoscale Design of an Ethylene Tetraaniline–Bipyridine-Based Crystalline Covalent Organic Framework for Enhanced Hydrogen Evolution Reaction

Satadal Sahoo, [a] Ishita Mukherjee, [a] Nitesh Kumar, [a] Sanhita Maity, [b] A.D. Dinga Wonanke, [c] Matthew A. Addicoat, [c] Anirban Pradhan, [a],*

[a]Department of Chemistry, Birla Institute of Technology (BIT) – Mesra, Ranchi, Jharkhand, 835215, India, Email: anirbanpradhan@bitmesra.ac.in

[b]Department of Applied Sciences, Amity University, Ranchi, Jharkhand

[c]School of Science and Technology, Nottingham Trent University, Clifton Lane, Nottingham, NG11 8NS, UK.

Section	Contents	Page no
S1	General Information	S1-S2
S2	Experimental section	S3-S9
S3	Theoretical Calculation	S10-S18
S4	Computational Details	S19-S24
S5	Comparison Table	S24-S26
S6	References	S26-S29

Section S1:

General information: All reagents and solvents were used as received without further purification. The precursors 4,4′,4″,4‴-(ethene-1,1,2,2-tetrayl) tetraaniline (ETTA) and [3,3′-bipyridine]-6,6′-dicarbaldehyde (Bpy) were procured from BLD Pharma and 3A Senrise, respectively. Nickel nitrate hexahydrate ($Ni(NO_3)_2\cdot 6H_2O$) was obtained from Spectrochem. The solvents methanol, n-butanol, and acetic acid were purchased from Qualigens and Thermo Fisher Scientific Pvt. Ltd., while o-dichlorobenzene was purchased from Spectrochem.

All electrochemical measurements were performed using an Autolab 302N electrochemical analyser potentiostat-galvanostat. The experiments were carried out in a conventional three-

electrode configuration with 0.5 M H_2SO_4 as the supporting electrolyte. A glassy carbon (GC) electrode was used as the working electrode. The catalyst ink was prepared by dispersing 2 mg of COF in a mixture containing 0.5 mL of water, 0.5 mL of isopropanol (IPA), and 10 µL of 0.5 wt% Nafion solution as the binder. Subsequently, one to two drops of the homogeneous suspension were uniformly cast onto the surface of the GC electrode and allowed to dry under ambient conditions. A standard Ag/AgCl electrode served as the reference electrode, while a platinum wire was used as the counter electrode to complete the electrochemical circuit for acidic hydrogen evolution reaction (HER) measurements.

Linear sweep voltammetry (LSV) was conducted at a scan rate of 10 mV s^{-1} at 27 °C. All measured potentials were converted to the reversible hydrogen electrode (RHE) scale according to the following equation:

$$E_{RHE} = E_{Ag/AgCl} + E^{\circ}_{Ag/AgCl} + 0.059 \times pH \text{..............................} (1)$$

Since 0.5 M H_2SO_4 was used as the electrolyte, the pH of the solution is approximately 0. Therefore, the potential conversion equation from the Ag/AgCl reference electrode to the reversible hydrogen electrode (RHE) scale can be simplified as follows:

$$E_{RHE} = E_{Ag/AgCl} + E^{\circ}_{Ag/AgCl} \text{..} (2)$$

where $E^{\circ}_{Ag/AgCl}$ is the standard potential of the Ag/AgCl reference electrode (0.197 V at 25 °C for saturated KCl). Hence, the final equation becomes:

$$E_{RHE} = E_{Ag/AgCl} + 0.197 \text{ V} \text{..} (3)$$

The overpotential is denoted by the symbol η. The overpotential values for both the pristine COF and its Ni-incorporated analogue were calculated at a benchmark current density of 10 mA cm^{-2} using the following relation:

$$\eta_{10} \text{ (HER)} = (0 - E_{RHE}) \text{ V vs RHE} \text{.................} (4)$$

To investigate the reaction kinetics, the Tafel slopes were determined by plotting the overpotential (η) as a function of the logarithm of the current density (log *j*), according to the Tafel equation:

$$\eta = a + b\log\left(\frac{i}{i_0}\right) \qquad (5)$$

where η represents the overpotential, *a* is a constant, *b* is the Tafel slope, *i* is the measured current density, and i_0 is the exchange current density.

Section S2:

Experimental section:

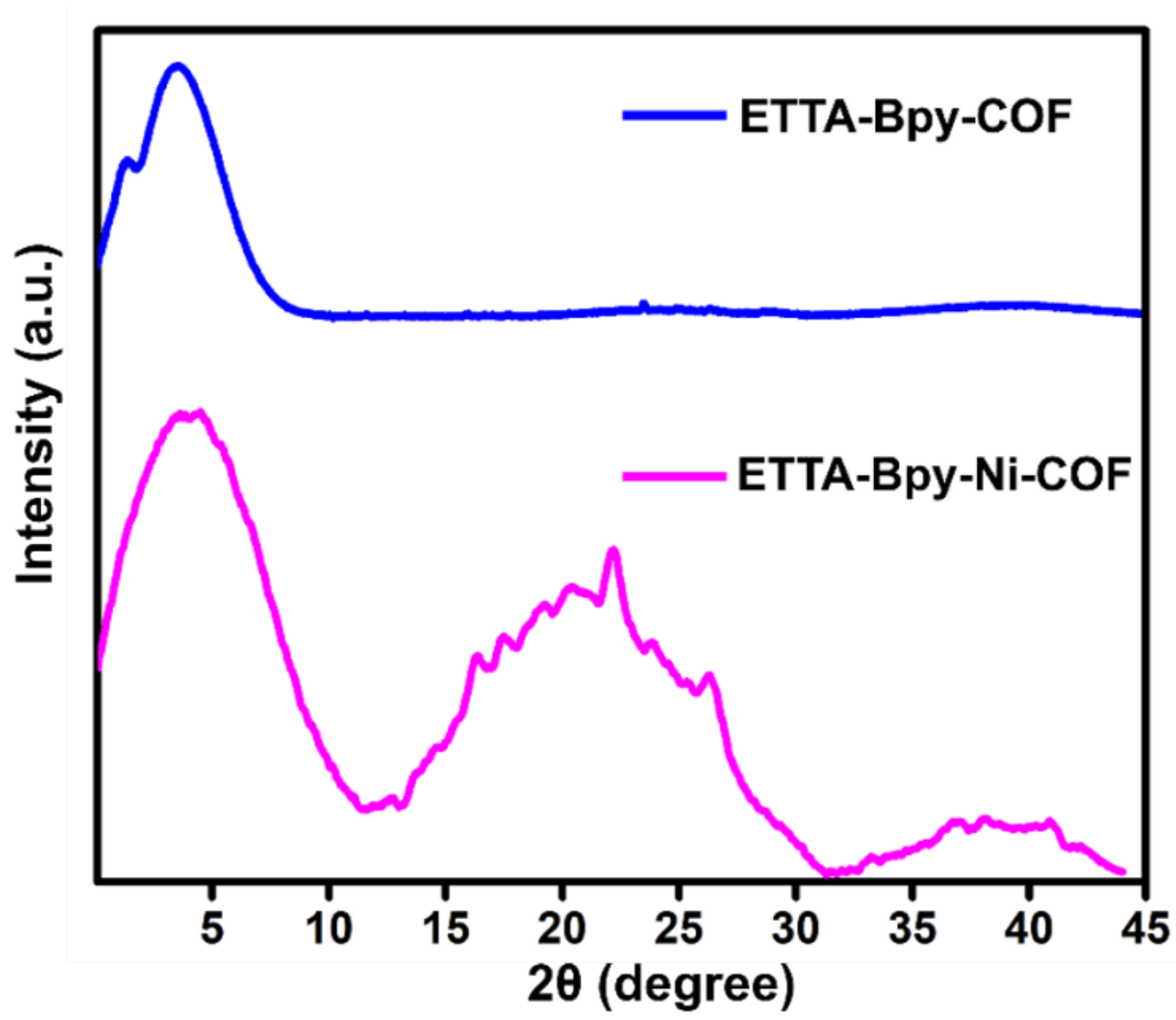

Fig S1. PXRD pattern of ETTA-Bpy-COFand ETTA-Bpy-Ni-COF.

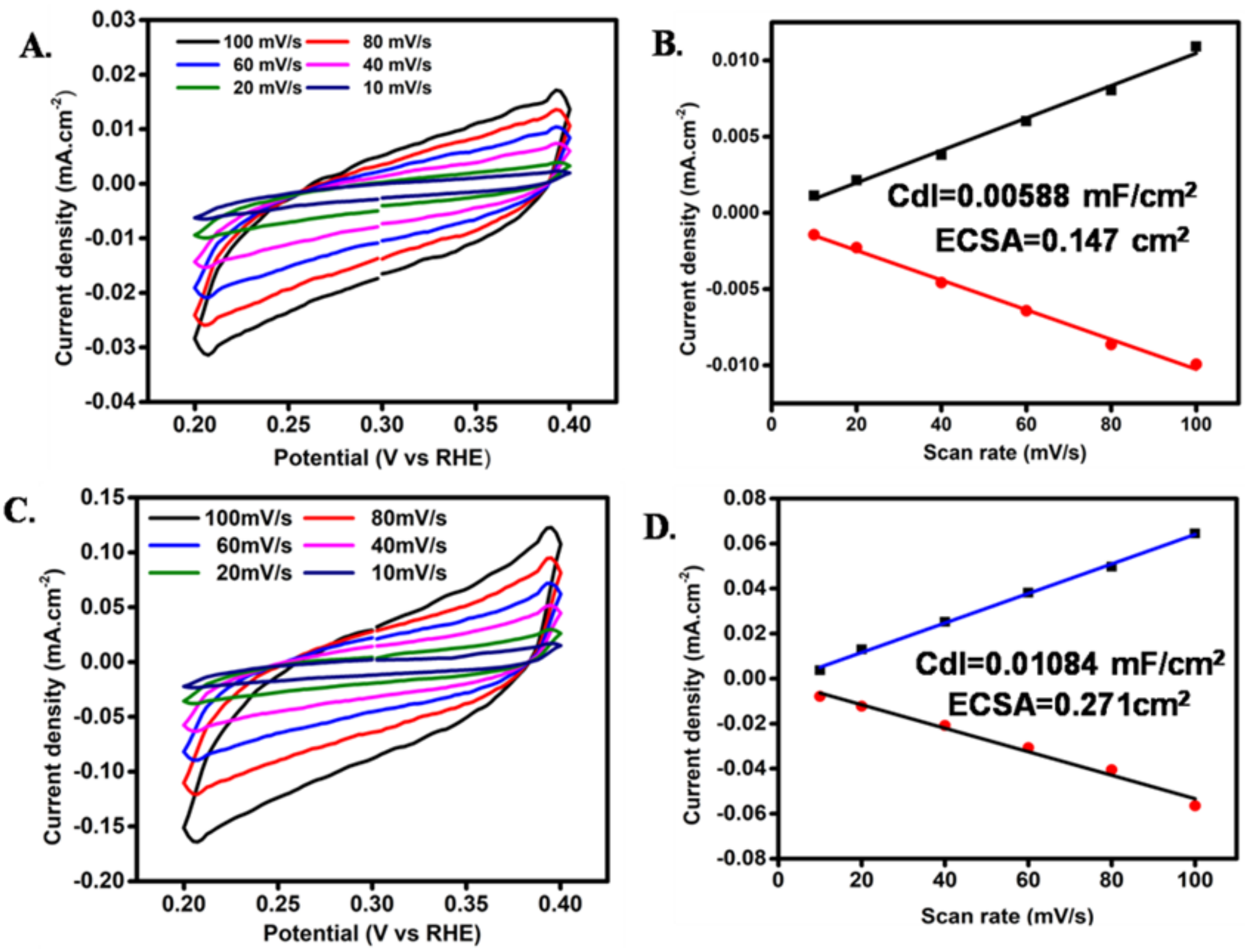

Fig S2: (A) and (C) are CV plot at different scan rate in 0.5M H_2SO_4,(B) and (D) are anodic and cathodic current vs scan rate plot of ETTA-Bpy-COF and ETTA-Bpy- Ni COF respectively.

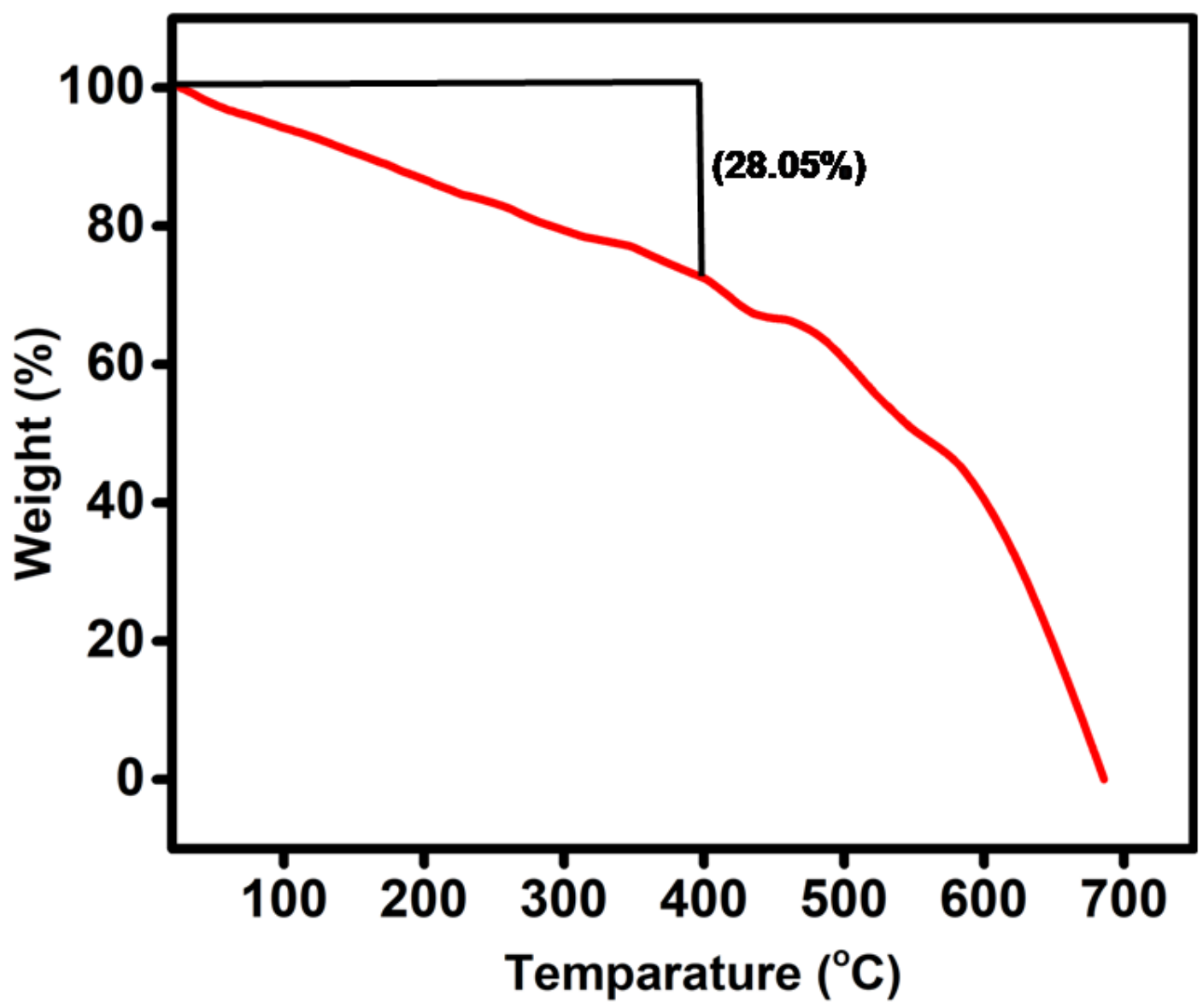

Fig S3: TGA plot of ETTA-Bpy-COF.

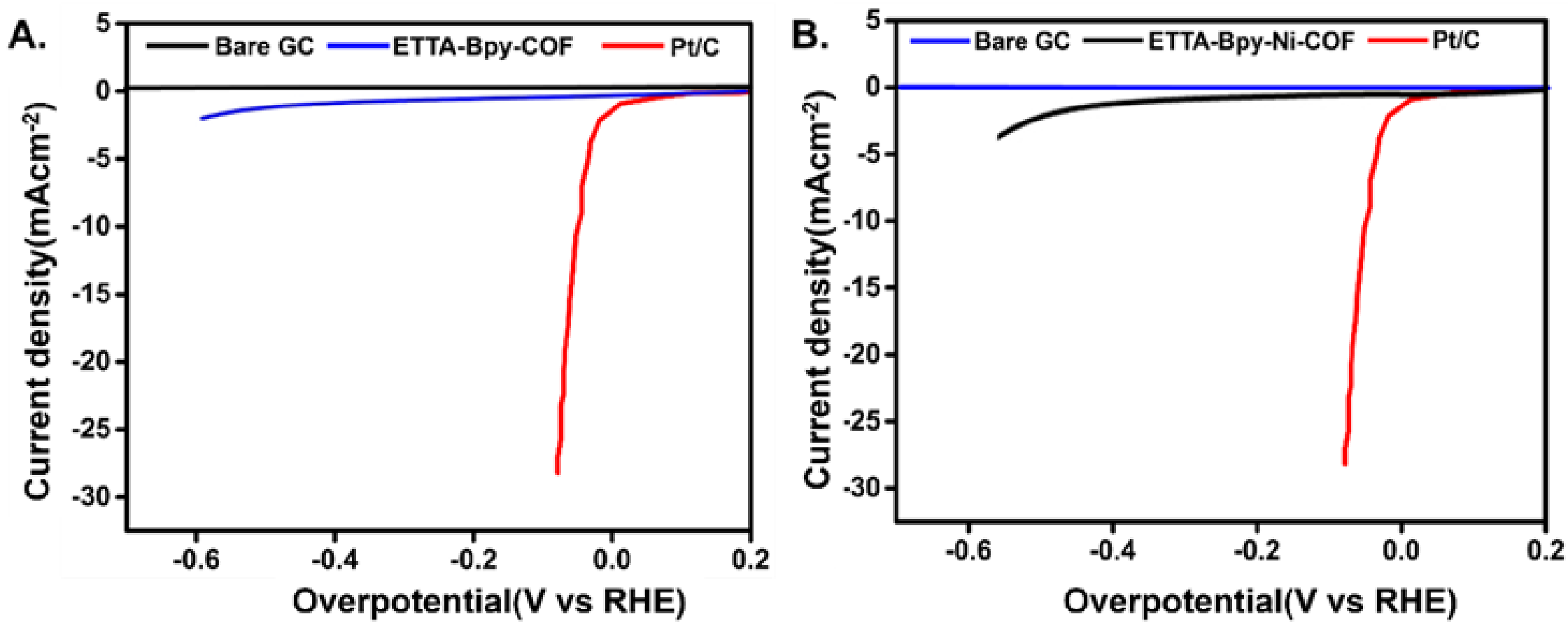

Fig S4: LSV of ETTA-Bpy-COF and ETTA-Bpy-Ni COF in 0.5 (M) NaOH solution.

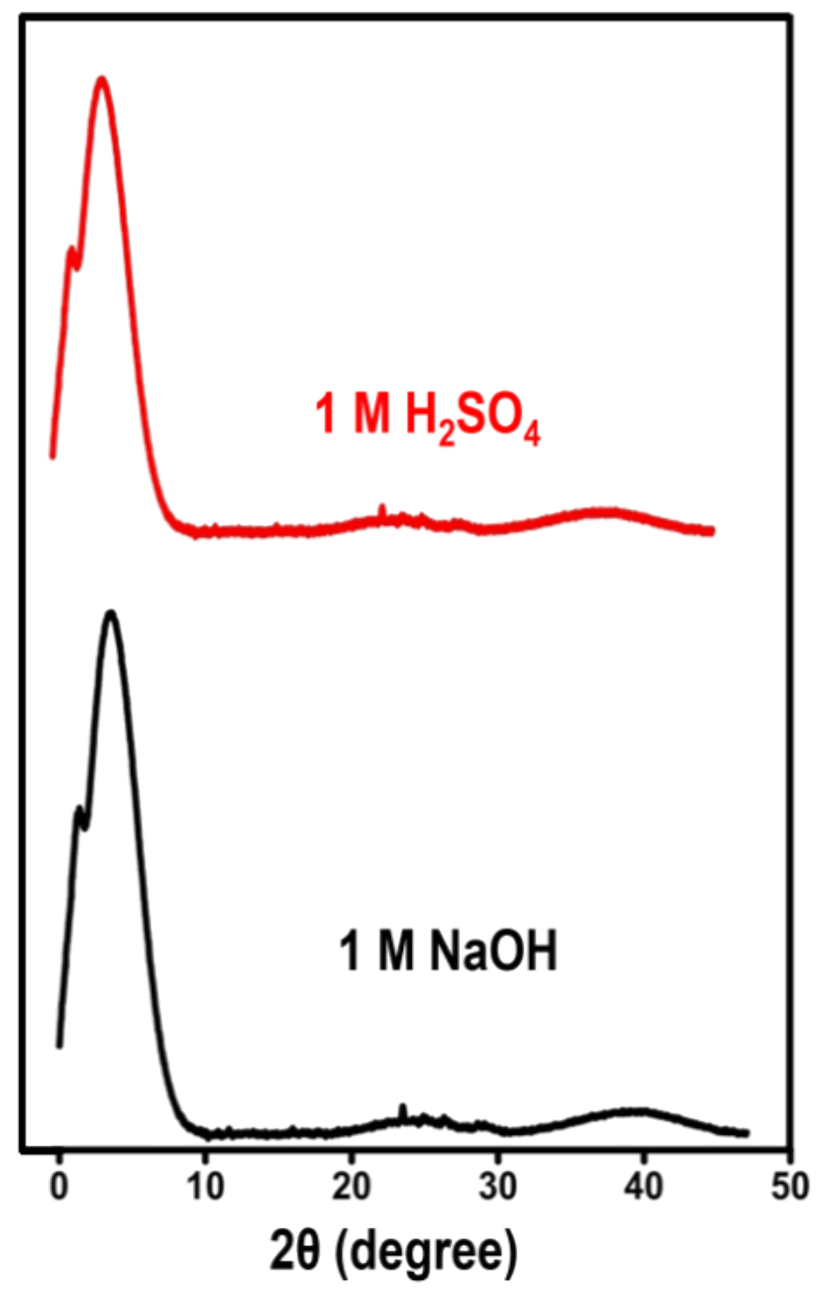

Fig S5: PXRD of ETTA-Bpy-COF indifferent conditions; 1 M H_2SO_4 and 1 M NaOH solution.

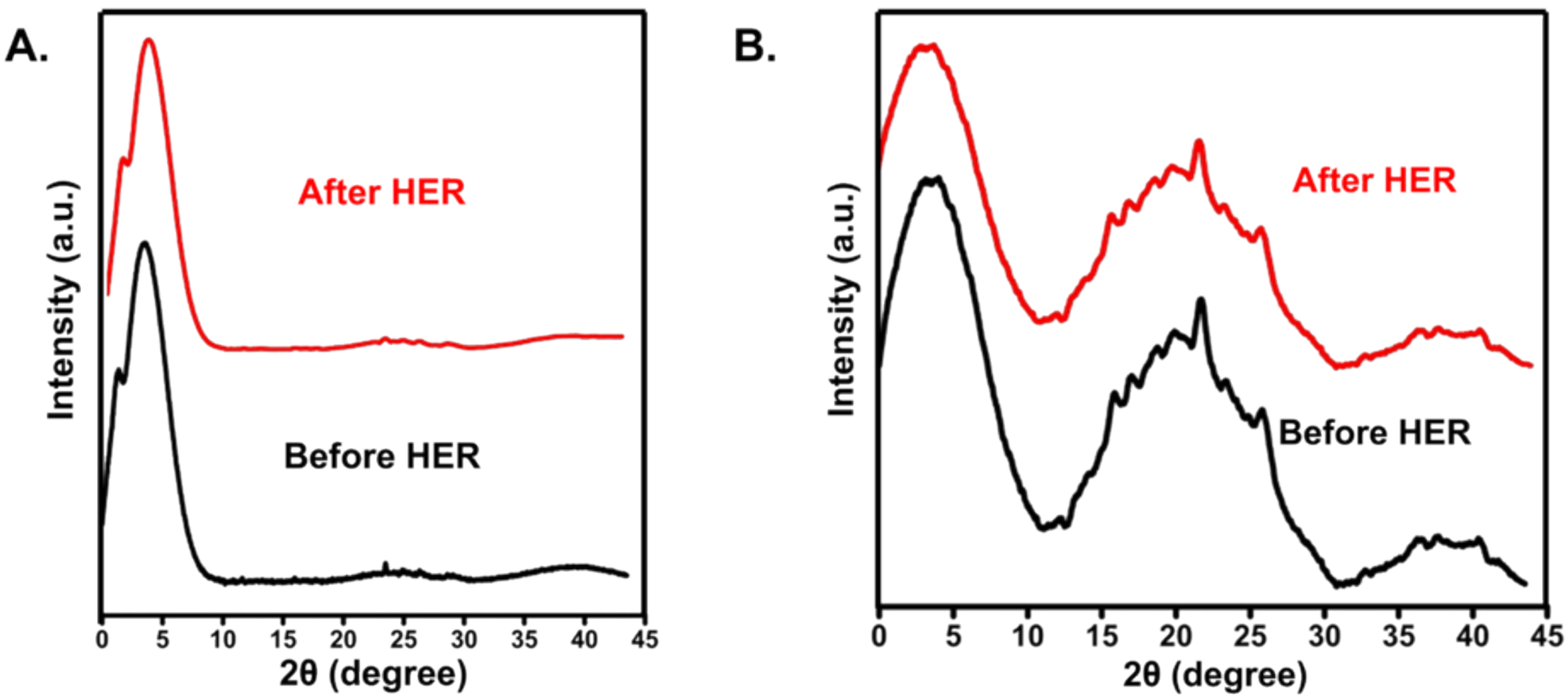

Fig S6: PXRD of ETTA-Bpy-COF (A) and ETTA-Bpy- Ni COF (B) before and after HER.

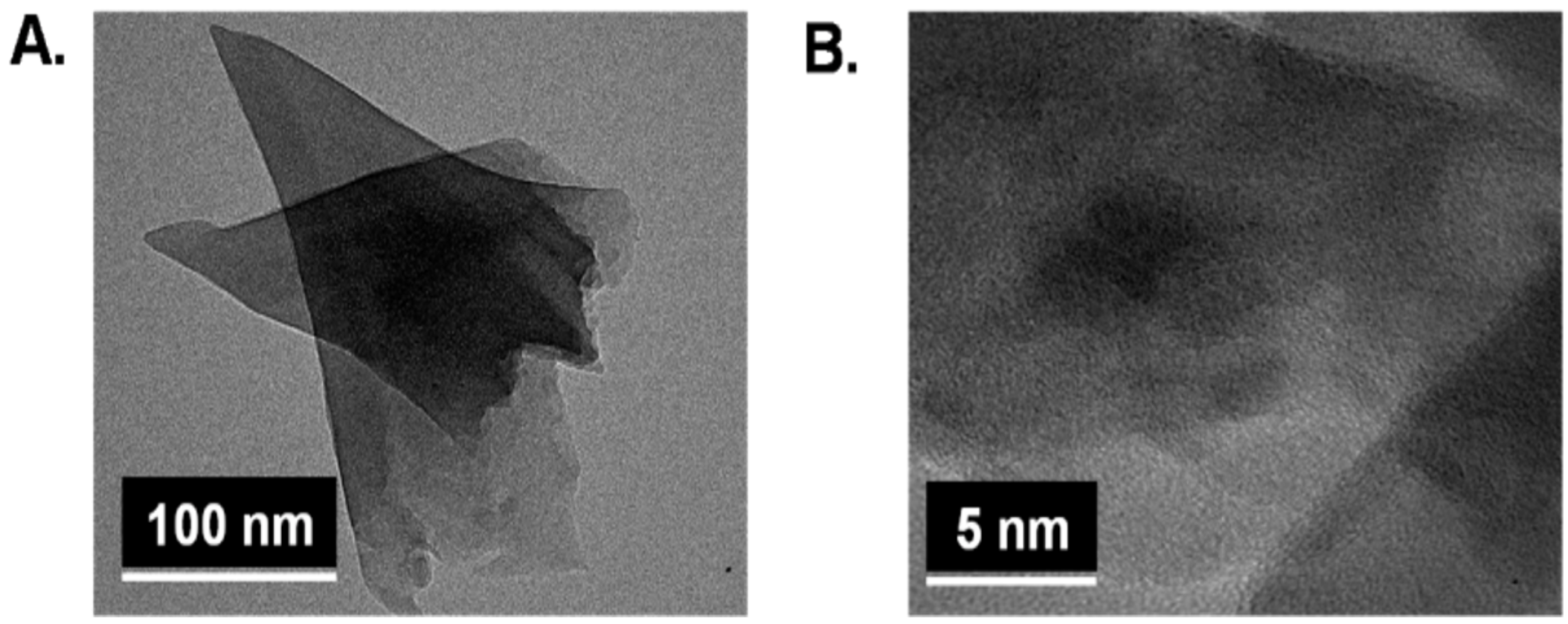

Fig S7: (A.) and (B.) are HRTEM image of ETTA-Bpy-COF before after HER.

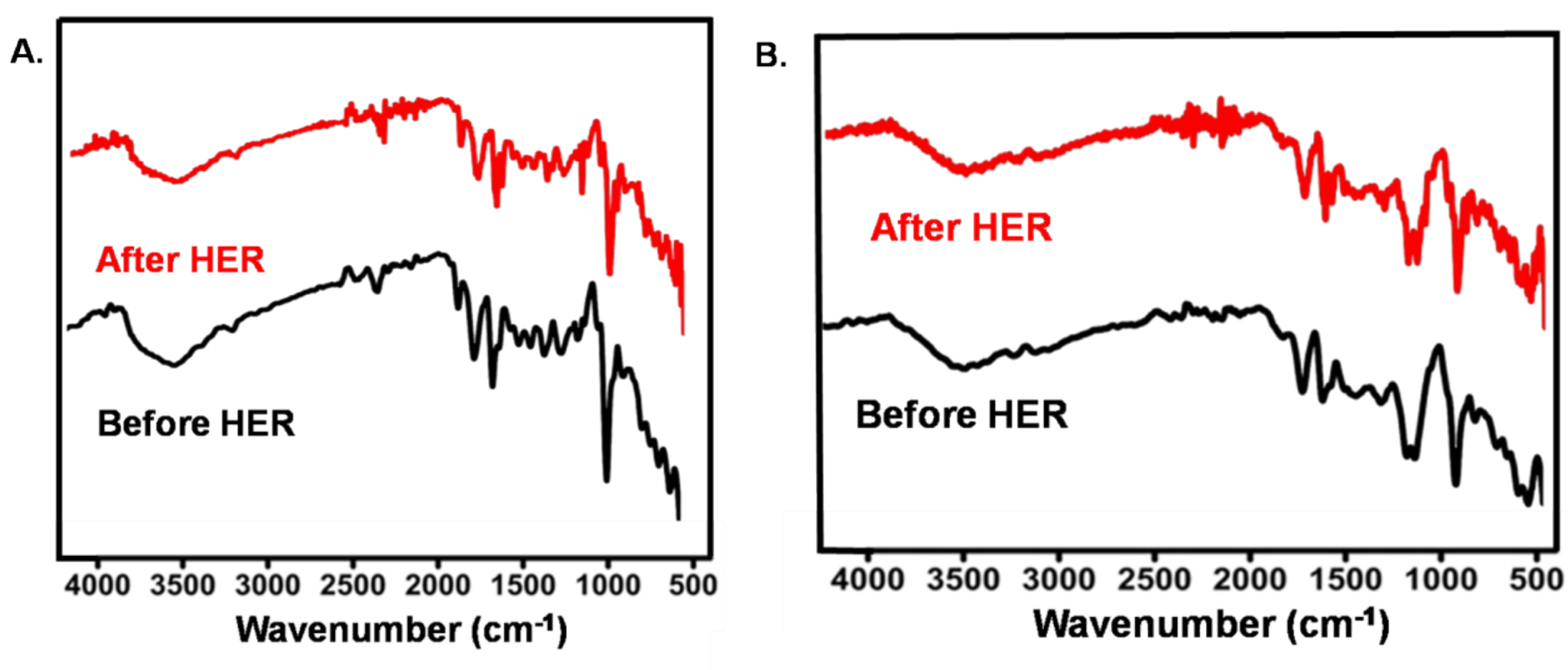

Fig S8: (A.) and (B.) are FTIR image of ETTA-Bpy-COF before after HER.

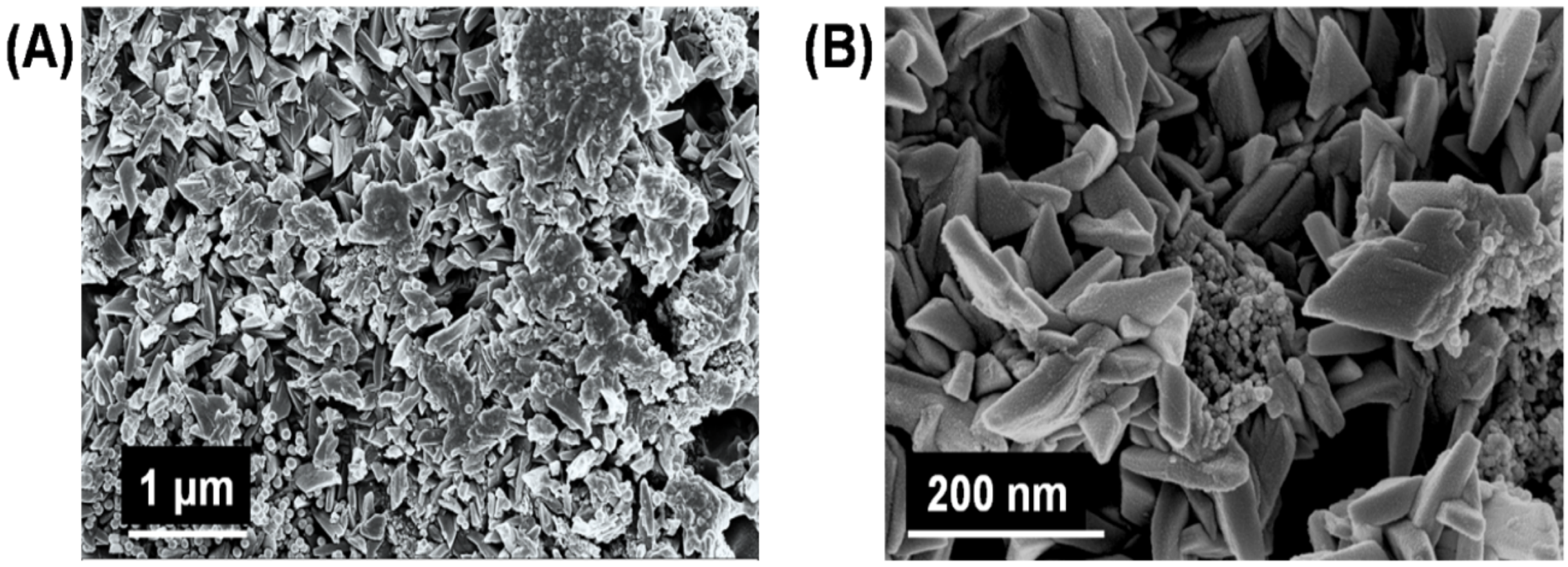

Fig S9: (A) SEM image of ETTA-Bpy-Ni COF before HER and (B) SEM imageof ETTA-Bpy-Ni COF after HER.

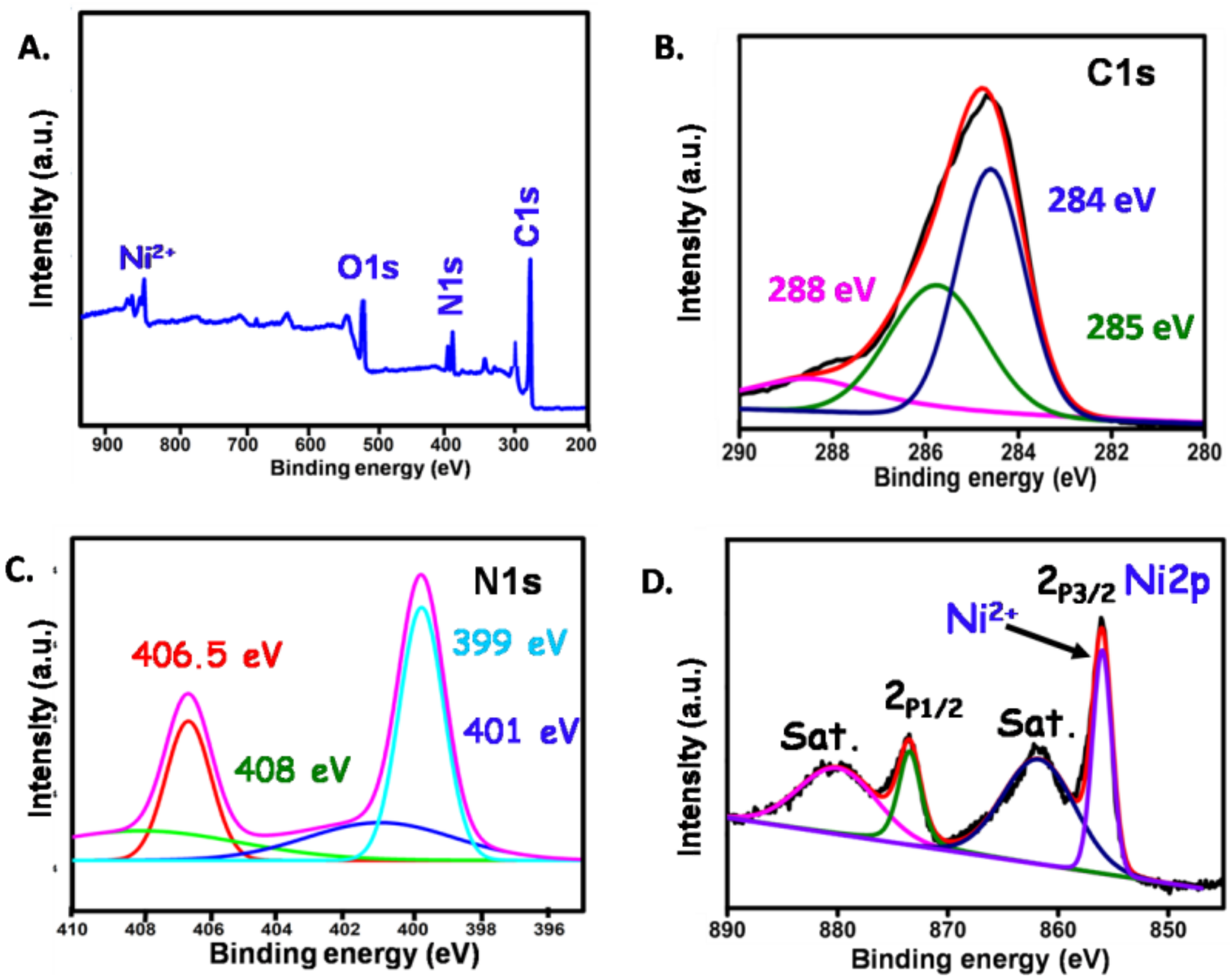

Fig S10: (A.) XPS survey spectra of ETTA-Bpy-COF,(B.) XPS survey spectra of ETTA-Bpy-Ni COF, (C) & (D) are deconvoluted spectra of C1s and N1s for ETTA-Bpy-COF.

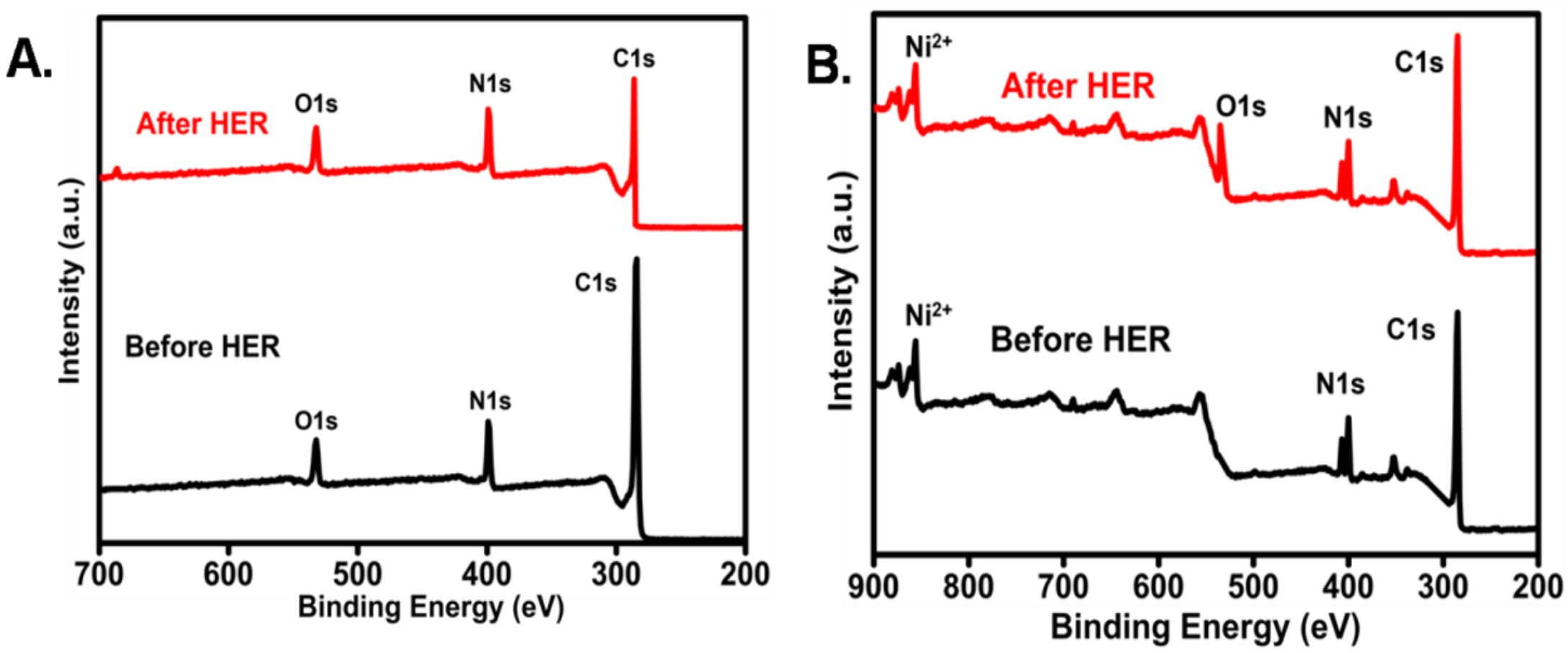

Fig S11: XPS spectraof ETTA-Bpy-COF (A) and ETTA-Bpy- Ni COF (B) before and after HER.

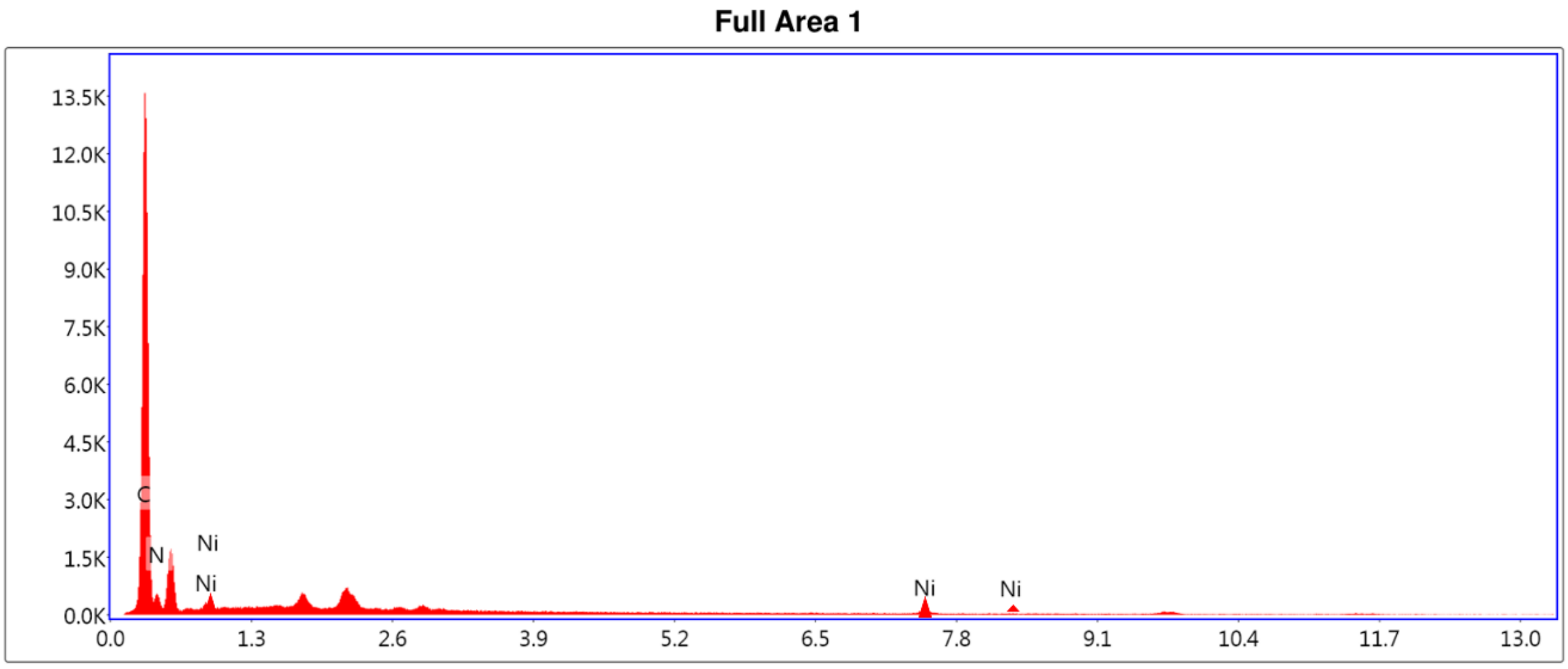

Fig S12: EDAX image of ETTA-Bpy-Ni COF.

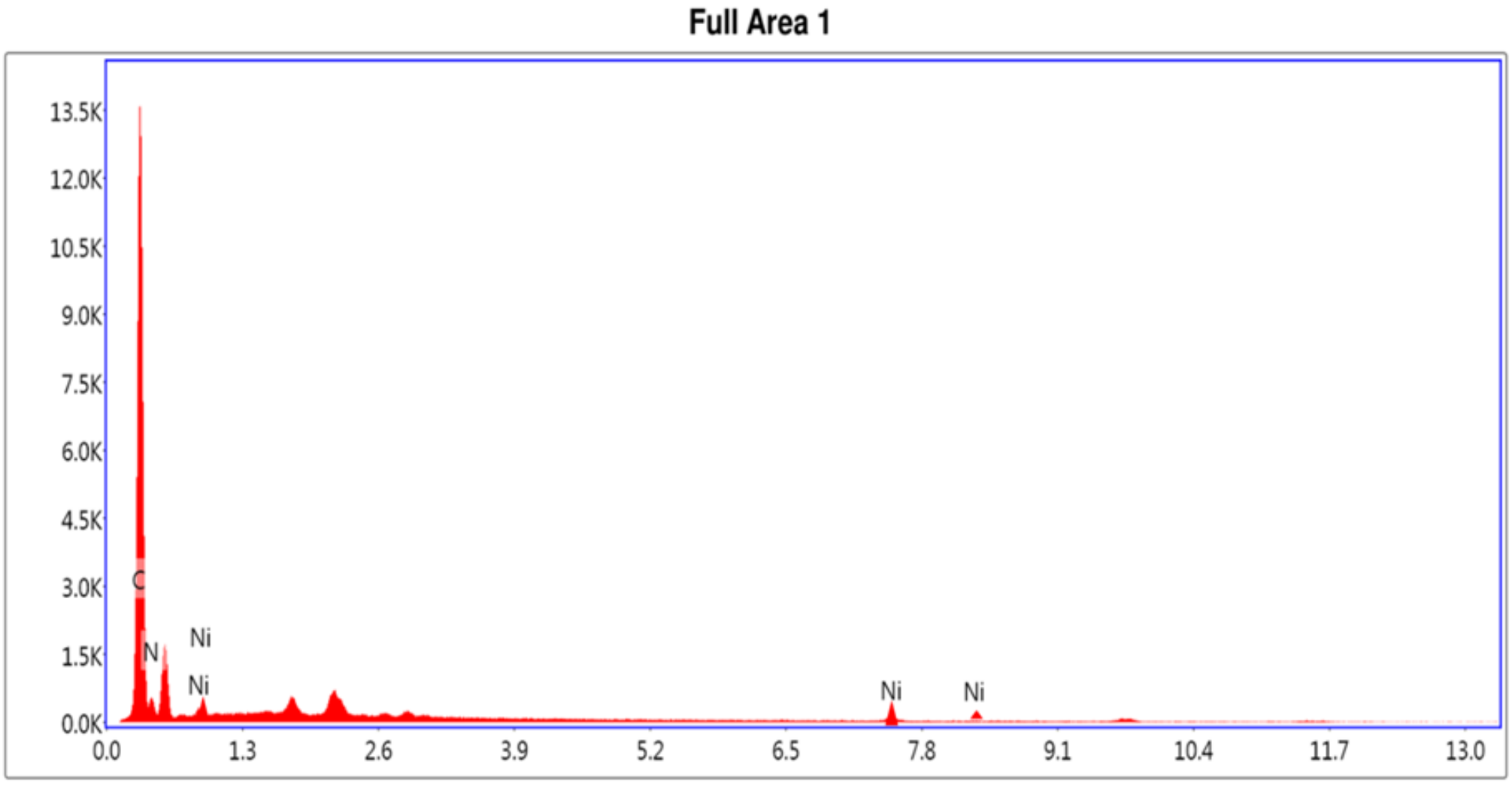

Fig S13: EDAX image of ETTA-Bpy-Ni COF after HER.

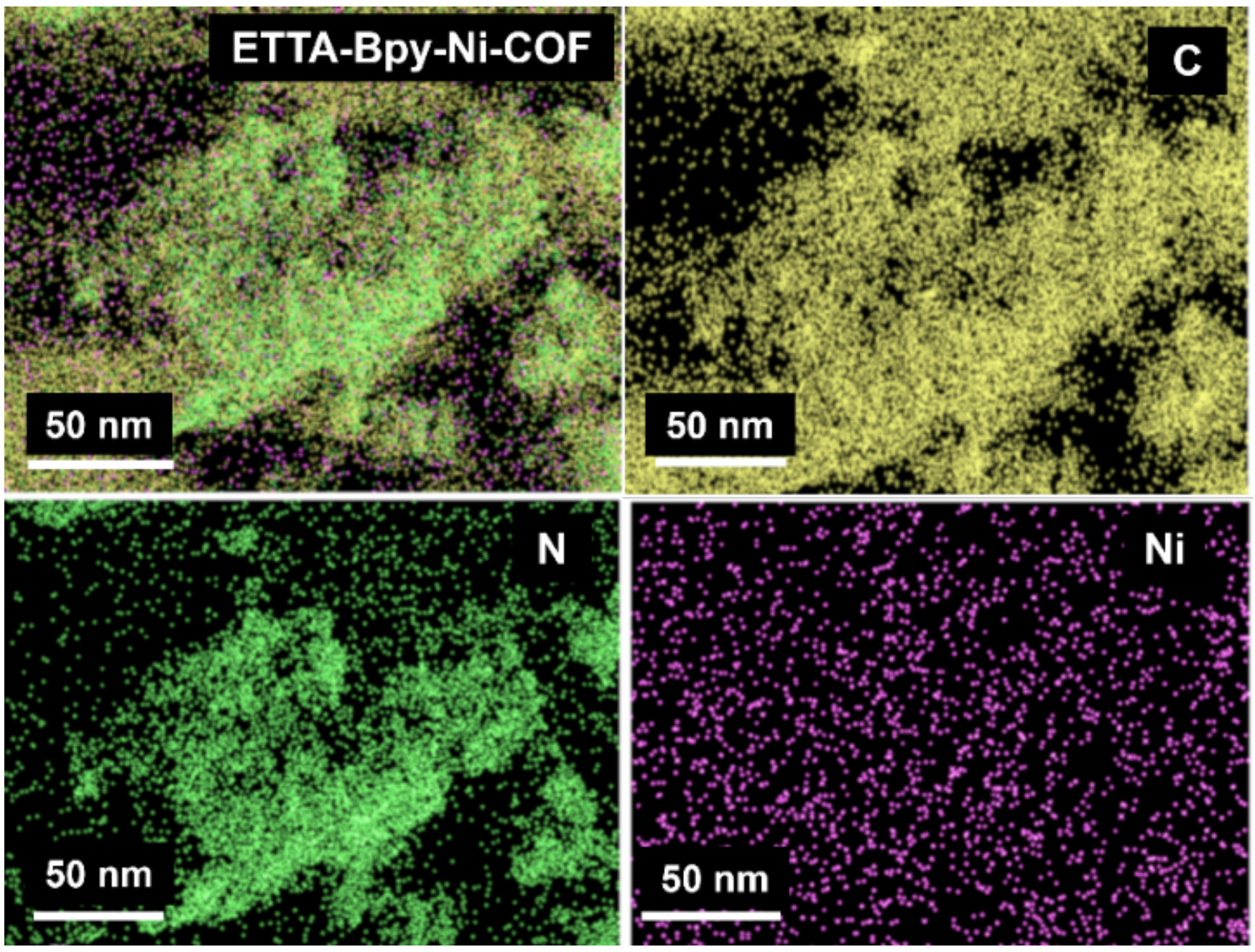

Fig S14: Elemental mapping of ETTA-Bpy-Ni-COF confirming the presence of C, N and Ni atoms.

Table S1. The weight content of Ni in ETTA-Bpy-COF.

Sample Name	Test element	Test solution element concentration C_o (mg/L)	Sample element content C_x(mg/kg)	Sample element content W (%)
**ETTA-Bpy-Ni-COF**	Ni	0.259	6475	0.6475
**ETTA-Bpy-Ni-COF (After HER)**	Ni	0.257	6425	0.6425

Section S3:

Theoretical Calculations: Computational details of powder X-ray diffraction for ETTA-Bpy-COF. Atomic positions and lattice coordinates of porous ETTA-Bpy-COF layers were optimized using the Self-Consistent-Charge Density-Functional Tight-Binding (SCC-DFTB) method as implemented in DFTB+ version commit fd31d87. All pair wise atomic interactions (C, N, H) were treated using the 30b-3-1 parameter and dispersion was accounted for using DFTD3 dispersion. It is known that the layer stacking is affected by the Coulomb repulsion between the partial atomic charges in adjacent layers. Hence, several slipped AA stacking possibilities were considered for ETTA-Bpy-COF by shifting adjacent layers with respect to each other indifferent directions (x, xy, y) up to 20 Å with a step size of 0.5 Å. All starting geometries were fully optimized without constraints (P1 space group). The predicted slipped structure has the lowest energy, while maintaining agreement with the experimental PXRD pattern.

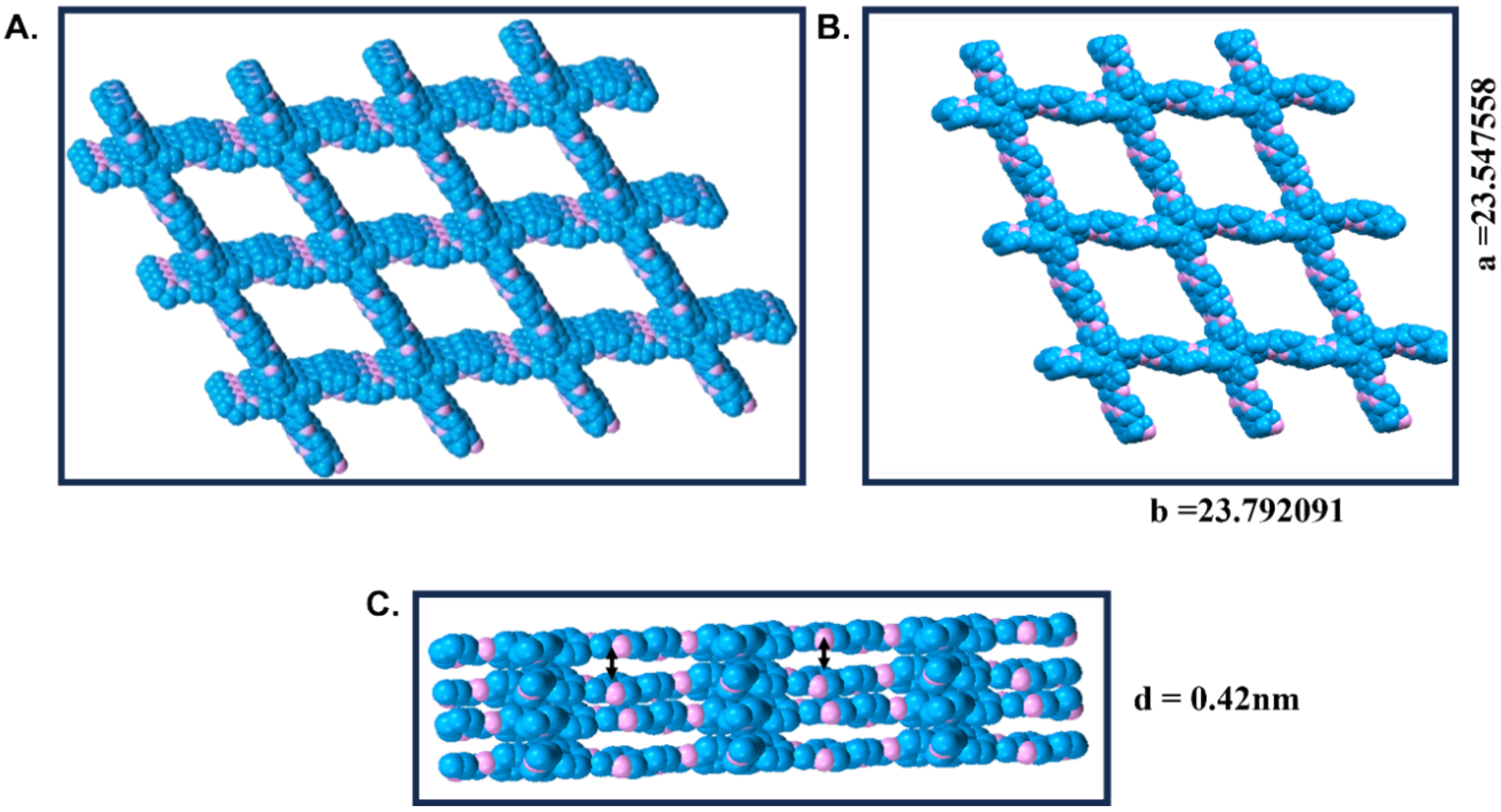

Fig S15: (A, B) Front view of Slipped XY stacking of ETTA-Bpy-COF, (C)Sideview of Slipped XY stacking of ETTA-Bpy-COF.

Table S2: Energies, stabilization energies, interlayer distances and band gaps of optimized **ETTA-Bpy-COF** structures.

	Interlayer distance Å	Total Energy (a.u.)	LJ energy (a.u.)	Per layer stabilization (kJ/mol)	HOMO-LUMO gap (eV)
monolayer		-116.7320226	-0.15140678		1.9659
AA	4.475	-233.5468	-0.4141371198	-108.735864	1.3356
slipXY	4.399	-233.5715	-0.4437759235	-141.06686	1.2315
AB	4.818	-233.577	-0.4538445833	-149.489329	1.2683

Unit cell structure and parameters

Lattice type - Triclinic

Space group - P1

a =23.547558; **b** =23.792091; **c** =9.614005;

α=88.927778°; β= 91.530137°; γ= 68.961173°.

Table S3: Fractional atomic coordinates for the unit cell of ETTA-Bpy-COF.

Number	Label	Charge	Sybyl Type	X frac + ESD	Y frac + ESD	Z frac + ESD	
1	C1	0	C.2	0.38539	0.39250	0.91851	x,y,z
2	C1	0	C.2	0.33398	0.44407	0.91458	x,y,z
3	C1	0	C.2	0.44780	0.39557	0.92151	x,y,z
4	C1	0	C.2	0.46528	0.42797	1.01690	x,y,z
5	C1	0	C.2	0.48756	0.36629	0.82465	x,y,z
6	C1	0	C.2	0.52035	0.43353	1.01030	x,y,z
7	H2	0	H	0.43453	0.45080	1.09666	x,y,z
8	C1	0	C.2	0.54297	0.37084	0.81939	x,y,z
9	H2	0	H	0.47443	0.33972	0.74948	x,y,z
10	C1	0	C.2	0.55976	0.40493	0.91224	x,y,z
11	H2	0	H	0.53295	0.46113	1.08427	x,y,z
12	H2	0	H	0.57246	0.34818	0.73761	x,y,z
13	C1	0	C.2	0.33792	0.50562	0.90738	x,y,z
14	C1	0	C.2	0.37602	0.52041	0.81496	x,y,z
15	C1	0	C.2	0.30308	0.55008	0.99609	x,y,z
16	C1	0	C.2	0.38410	0.57579	0.82259	x,y,z
17	H2	0	H	0.40059	0.48850	0.73926	x,y,z

18	C1	0	C.2	0.31021	0.60586	1.00237	x,y,z
19	H2	0	H	0.27060	0.54119	1.06161	x,y,z
20	C1	0	C.2	0.35151	0.61901	0.91612	x,y,z
21	H2	0	H	0.41557	0.58530	0.75436	x,y,z
22	H2	0	H	0.28404	0.63751	1.07660	x,y,z
23	C1	0	C.2	0.38338	0.33014	0.91417	x,y,z
24	C1	0	C.2	0.42581	0.28305	0.99525	x,y,z
25	C1	0	C.2	0.34247	0.31604	0.82508	x,y,z
26	C1	0	C.2	0.42432	0.22480	0.99527	x,y,z
27	H2	0	H	0.45928	0.29168	1.06225	x,y,z
28	C1	0	C.2	0.34056	0.25801	0.82567	x,y,z
29	H2	0	H	0.31147	0.34994	0.75598	x,y,z
30	C1	0	C.2	0.38099	0.21198	0.91110	x,y,z
31	H2	0	H	0.45592	0.19080	1.06414	x,y,z
32	H2	0	H	0.30732	0.24883	0.75899	x,y,z
33	C1	0	C.2	0.27086	0.44283	0.92092	x,y,z
34	C1	0	C.2	0.25582	0.40926	1.01888	x,y,z
35	C1	0	C.2	0.22700	0.47704	0.82826	x,y,z
36	C1	0	C.2	0.19890	0.40803	1.02045	x,y,z
37	H2	0	H	0.28929	0.38250	1.09550	x,y,z
38	C1	0	C.2	0.17015	0.47581	0.82958	x,y,z
39	H2	0	H	0.23744	0.50495	0.75005	x,y,z
40	C1	0	C.2	0.15564	0.44108	0.92598	x,y,z
41	H2	0	H	0.18817	0.38001	1.09784	x,y,z
42	H2	0	H	0.13726	0.50191	0.75043	x,y,z

43	N3	0	N.2	0.61729	0.41340	0.90761	x,y,z
44	N3	0	N.2	0.36214	0.67496	0.92030	x,y,z
45	N3	0	N.2	0.37457	0.15410	0.91363	x,y,z
46	N3	0	N.2	0.09650	0.43597	0.92665	x,y,z
47	C1	0	C.2	0.77489	0.35383	0.79465	x,y,z
48	C1	0	C.2	0.82768	0.36774	0.80020	x,y,z
49	C1	0	C.2	0.82671	0.42191	0.85732	x,y,z
50	C1	0	C.2	0.77157	0.46139	0.90741	x,y,z
51	C1	0	C.2	0.72114	0.39399	0.84795	x,y,z
52	H2	0	H	0.77604	0.31203	0.74986	x,y,z
53	H2	0	H	0.86909	0.33655	0.75862	x,y,z
54	H2	0	H	0.76905	0.50348	0.95280	x,y,z
55	C1	0	C.2	0.88259	0.43697	0.86456	x,y,z
56	C1	0	C.2	0.93867	0.39376	0.91073	x,y,z
57	C1	0	C.2	0.88094	0.49448	0.82382	x,y,z
58	H2	0	H	0.94164	0.34912	0.94462	x,y,z
59	C1	0	C.2	0.93424	0.50751	0.82732	x,y,z
60	H2	0	H	0.83872	0.52881	0.78626	x,y,z
61	C1	0	C.2	0.98922	0.46305	0.87379	x,y,z
62	H2	0	H	0.93261	0.55175	0.79332	x,y,z
63	C1	0	C.2	0.66550	0.37930	0.84586	x,y,z
64	H2	0	H	0.66640	0.33861	0.79583	x,y,z
65	C1	0	C.2	0.04614	0.47549	0.87450	x,y,z
66	H2	0	H	0.04509	0.51823	0.83144	x,y,z
67	N3	0	N.2	0.72090	0.44651	0.90239	x,y,z

68	N3	0	N.2	0.98991	0.40766	0.91444	x,y,z
69	C1	0	C.2	0.39817	0.77945	0.91812	x,y,z
70	C1	0	C.2	0.41002	0.83298	0.91583	x,y,z
71	C1	0	C.2	0.36878	0.88537	0.97221	x,y,z
72	C1	0	C.2	0.31610	0.88201	1.03129	x,y,z
73	C1	0	C.2	0.34481	0.77833	0.97777	x,y,z
74	H2	0	H	0.43022	0.73959	0.87336	x,y,z
75	H2	0	H	0.45080	0.83373	0.86774	x,y,z
76	H2	0	H	0.28362	0.92094	1.07802	x,y,z
77	C1	0	C.2	0.37990	0.94316	0.96784	x,y,z
78	C1	0	C.2	0.43620	0.94450	0.99522	x,y,z
79	C1	0	C.2	0.33385	0.99599	0.93604	x,y,z
80	H2	0	H	0.47419	0.90354	1.02229	x,y,z
81	C1	0	C.2	0.34423	0.04813	0.93191	x,y,z
82	H2	0	H	0.28825	0.99704	0.91233	x,y,z
83	C1	0	C.2	0.40116	0.04740	0.96055	x,y,z
84	H2	0	H	0.30679	0.08986	0.90652	x,y,z
85	C1	0	C.2	0.32950	0.72342	0.98259	x,y,z
86	C1	0	C.2	0.41481	0.10334	0.96014	x,y,z
87	H2	0	H	0.45953	0.10070	0.99693	x,y,z
88	N3	0	N.2	0.30566	0.82941	1.03264	x,y,z
89	N3	0	N.2	0.44510	0.99586	0.99150	x,y,z
90	H2	0	H	0.28885	0.72569	1.03536	x,y,z
91	C1	0	C.3	-0.01078	0.46305	0.87379	-1+x,y,z
92	C1	0	C.3	1.04614	0.47549	0.87450	1+x,y,z

93	C1	0	C.2	0.33385	-0.00401	0.93604	x,-1+y,z
94	C1	0	C.2	0.34423	1.04813	0.93191	x,1+y,z
95	C1	0	C.2	0.40116	1.04740	0.96055	x,1+y,z
96	N3	0	N.pl3	0.44510	-0.00414	0.99150	x,-1+y,z
97	C1	0	C.2	0.43927	0.42988	0.41758	x,y,z
98	C1	0	C.2	0.38856	0.48167	0.41968	x,y,z
99	C1	0	C.2	0.50198	0.43139	0.43467	x,y,z
100	C1	0	C.2	0.51671	0.46545	0.53811	x,y,z
101	C1	0	C.2	0.54803	0.39673	0.34866	x,y,z
102	C1	0	C.2	0.57553	0.46664	0.55085	x,y,z
103	H2	0	H	0.48285	0.49127	0.60883	x,y,z
104	C1	0	C.2	0.60669	0.39782	0.36143	x,y,z
105	H2	0	H	0.53833	0.36925	0.26981	x,y,z
106	C1	0	C.2	0.62097	0.43283	0.46319	x,y,z
107	H2	0	H	0.58585	0.49386	0.62957	x,y,z
108	H2	0	H	0.64001	0.37250	0.28900	x,y,z
109	C1	0	C.2	0.39278	0.54269	0.40201	x,y,z
110	C1	0	C.2	0.42933	0.55446	0.30052	x,y,z
111	C1	0	C.2	0.35647	0.59070	0.48075	x,y,z
112	C1	0	C.2	0.43128	0.61205	0.28222	x,y,z
113	H2	0	H	0.45659	0.51921	0.23553	x,y,z
114	C1	0	C.2	0.35809	0.64841	0.46197	x,y,z
115	H2	0	H	0.32676	0.58346	0.55738	x,y,z
116	C1	0	C.2	0.39551	0.65950	0.36172	x,y,z
117	H2	0	H	0.46063	0.61984	0.20534	x,y,z

118	H2	0	H	0.33118	0.68359	0.52835	x,y,z
119	C1	0	C.2	0.43510	0.36900	0.39802	x,y,z
120	C1	0	C.2	0.47574	0.31821	0.47148	x,y,z
121	C1	0	C.2	0.39160	0.36078	0.30586	x,y,z
122	C1	0	C.2	0.46986	0.26208	0.46251	x,y,z
123	H2	0	H	0.51222	0.32184	0.53692	x,y,z
124	C1	0	C.2	0.38412	0.30519	0.30085	x,y,z
125	H2	0	H	0.36230	0.39789	0.24068	x,y,z
126	C1	0	C.2	0.42346	0.25524	0.37722	x,y,z
127	H2	0	H	0.50114	0.22498	0.52443	x,y,z
128	H2	0	H	0.34855	0.30028	0.23414	x,y,z
129	C1	0	C.2	0.32572	0.48025	0.43099	x,y,z
130	C1	0	C.2	0.31361	0.43866	0.51994	x,y,z
131	C1	0	C.2	0.27748	0.52024	0.34840	x,y,z
132	C1	0	C.2	0.25658	0.43291	0.51398	x,y,z
133	H2	0	H	0.34888	0.40916	0.59015	x,y,z
134	C1	0	C.2	0.22008	0.51560	0.34525	x,y,z
135	H2	0	H	0.28443	0.55504	0.28540	x,y,z
136	C1	0	C.2	0.20940	0.47128	0.42785	x,y,z
137	H2	0	H	0.24835	0.39903	0.57815	x,y,z
138	H2	0	H	0.18514	0.54595	0.27610	x,y,z
139	N3	0	N.2	0.67977	0.43805	0.47401	x,y,z
140	N3	0	N.2	0.40140	0.71712	0.34445	x,y,z
141	N3	0	N.2	0.41455	0.19887	0.36481	x,y,z
142	N3	0	N.2	0.15176	0.46343	0.42842	x,y,z

143	C1	0	C.2	0.84019	0.36882	0.38890	x,y,z
144	C1	0	C.2	0.89252	0.38324	0.38445	x,y,z
145	C1	0	C.2	0.89083	0.44013	0.42506	x,y,z
146	C1	0	C.2	0.83601	0.48115	0.47256	x,y,z
147	C1	0	C.2	0.78626	0.41138	0.43514	x,y,z
148	H2	0	H	0.84150	0.32532	0.35420	x,y,z
149	H2	0	H	0.93380	0.35068	0.34562	x,y,z
150	H2	0	H	0.83323	0.52521	0.50596	x,y,z
151	C1	0	C.2	0.94511	0.45716	0.41341	x,y,z
152	C1	0	C.2	0.00066	0.41923	0.46048	x,y,z
153	C1	0	C.2	0.94049	0.51075	0.35351	x,y,z
154	H2	0	H	0.00622	0.37618	0.50980	x,y,z
155	C1	0	C.2	0.99042	0.52532	0.34097	x,y,z
156	H2	0	H	0.89690	0.54209	0.31364	x,y,z
157	C1	0	C.2	0.04496	0.48608	0.38927	x,y,z
158	H2	0	H	0.98635	0.56813	0.29234	x,y,z
159	C1	0	C.2	0.72990	0.39845	0.43203	x,y,z
160	H2	0	H	0.73077	0.35599	0.39167	x,y,z
161	C1	0	C.2	0.10010	0.50089	0.37772	x,y,z
162	H2	0	H	0.09631	0.54383	0.33083	x,y,z
163	N3	0	N.2	0.78565	0.46602	0.47628	x,y,z
164	N3	0	N.2	0.04847	0.43453	0.44755	x,y,z
165	C1	0	C.2	0.43225	0.82293	0.32876	x,y,z
166	C1	0	C.2	0.44429	0.87613	0.32853	x,y,z
167	C1	0	C.2	0.39887	0.93043	0.36285	x,y,z

168	C1	0	C.2	0.34134	0.92987	0.39548	x,y,z
169	C1	0	C.2	0.37461	0.82403	0.36560	x,y,z
170	H2	0	H	0.46787	0.78138	0.30238	x,y,z
171	H2	0	H	0.48905	0.87490	0.30146	x,y,z
172	H2	0	H	0.30534	0.97083	0.42364	x,y,z
173	C1	0	C.2	0.41207	0.98658	0.36906	x,y,z
174	C1	0	C.2	0.46633	0.98649	0.43337	x,y,z
175	C1	0	C.2	0.37049	0.04112	0.31298	x,y,z
176	H2	0	H	0.49923	0.94542	0.47928	x,y,z
177	C1	0	C.2	0.38322	0.09389	0.32216	x,y,z
178	H2	0	H	0.32856	0.04274	0.26079	x,y,z
179	C1	0	C.2	0.43772	0.09189	0.38892	x,y,z
180	H2	0	H	0.35093	0.13561	0.27735	x,y,z
181	C1	0	C.2	0.36110	0.76847	0.37668	x,y,z
182	C1	0	C.2	0.45298	0.14618	0.40485	x,y,z
183	H2	0	H	0.49721	0.14064	0.45023	x,y,z
184	N3	0	N.2	0.33093	0.87725	0.39736	x,y,z
185	N3	0	N.2	0.47754	0.03848	0.44199	x,y,z
186	H2	0	H	0.31718	0.77134	0.41383	x,y,z
187	C1	0	C.2	-0.05489	0.45716	0.41341	-1+x,y,z
188	C1	0	C.2	1.00066	0.41923	0.46048	1+x,y,z
189	C1	0	C.2	-0.00958	0.52532	0.34097	-1+x,y,z
190	C1	0	C.2	1.04496	0.48608	0.38927	1+x,y,z
191	C1	0	C.2	0.41207	-0.01342	0.36906	x,-1+y,z
192	C1	0	C.2	0.46633	-0.01351	0.43337	x,-1+y,z

193	C1	0	C.2	0.37049	1.04112	0.31298	x,1+y,z
194	N3	0	N.pl3	0.47754	1.03848	0.44199	x,1+y,z

Section S4:

Computational Details:

To investigate the hydrogen evolution reaction (HER) activity of the COFs, finite cluster models consisting of a single COF layer were used to reduce the computational cost associated with periodic calculations while preserving the local electronic environment around the active sites. To achieve this, the pristine COF and the Ni-doped COF were considered.

For each host system (pristine and Ni-doped), multiple hydrogen adsorption configurations were generated by placing a hydrogen atom at the different potential adsorption sites illustrated in **FigS20.** The adsorption sites correspond to the Ni centre, and the nitrogen atoms labelled 1-16. The resulting adsorption configurations are illustrated in **FigS23** and **S24** for the pristine and Ni-doped systems, respectively.

All geometry optimizations and vibrational frequency calculations were carried out using the ORCA[1,2,3]quantum chemistry package at the r^2SCAN-3c level of theory.[4,5] The r^2SCAN-3c method is a composite density functional theory (DFT) approach based on the meta-generalized gradient approximation (meta-GGA) r^2SCAN exchange–correlation functional combined with a compact triple-ζ Gaussian basis set (mTZVPP), semi-classical dispersion corrections, and basis-set error corrections. Specifically, the method incorporates the D4 London dispersion correction and the geometrical counterpoise (gCP) correction to reduce basis-set superposition errors arising from the use of moderate-sized basis sets.[1,5–13]

The r^2SCAN-3c composite density functional method was selected because it provides an excellent balance between computational efficiency and accuracy for large molecular systems containing approximately 270–273 atoms.[4] In addition, the method includes analytical gradients and efficient corrections for dispersion, basis-set incompleteness, and basis-set superposition effects, making it particularly suitable for large supramolecular and framework-based systems.[1,5–13]For the hydrogen-adsorbed configurations, a doublet spin multiplicity was employed due to the

presence of an unpaired electron associated with the adsorbed hydrogen atom, whereas singlet multiplicities were used for the pristine host structures and the isolated H_2 molecule.

The Gibbs free energy of hydrogen adsorption, ∆G_{H^*} was evaluated using equation (1).

$$\square G_{H^*} = G_{complex} - \left(G_{Host} + \frac{1}{2} G_{H_2}\right)$$

Where ∆$G_{complex}$ is the Gibbs free energy of the hydrogen-adsorbed configuration, G_{host} corresponds to the Gibbs free energy of the prestine or Ni-doped host system and G_{H_2} is the Gibbs free energy of an isolated hydrogen molecule. Charge redistribution upon hydrogen adsorption was analysed using the Quantum Atoms in Molecules (QTAIM) framework through Bader atom-in-molecule (AIM) analysis as implemented in the Amsterdam Modelling Suite (AMS) version AMS2026.1. [14]The electronic structure calculations were performed using the ADF engine at the r^2SCAN/TZ2P level of theory with Grimme D4 dispersion corrections. [15–17]

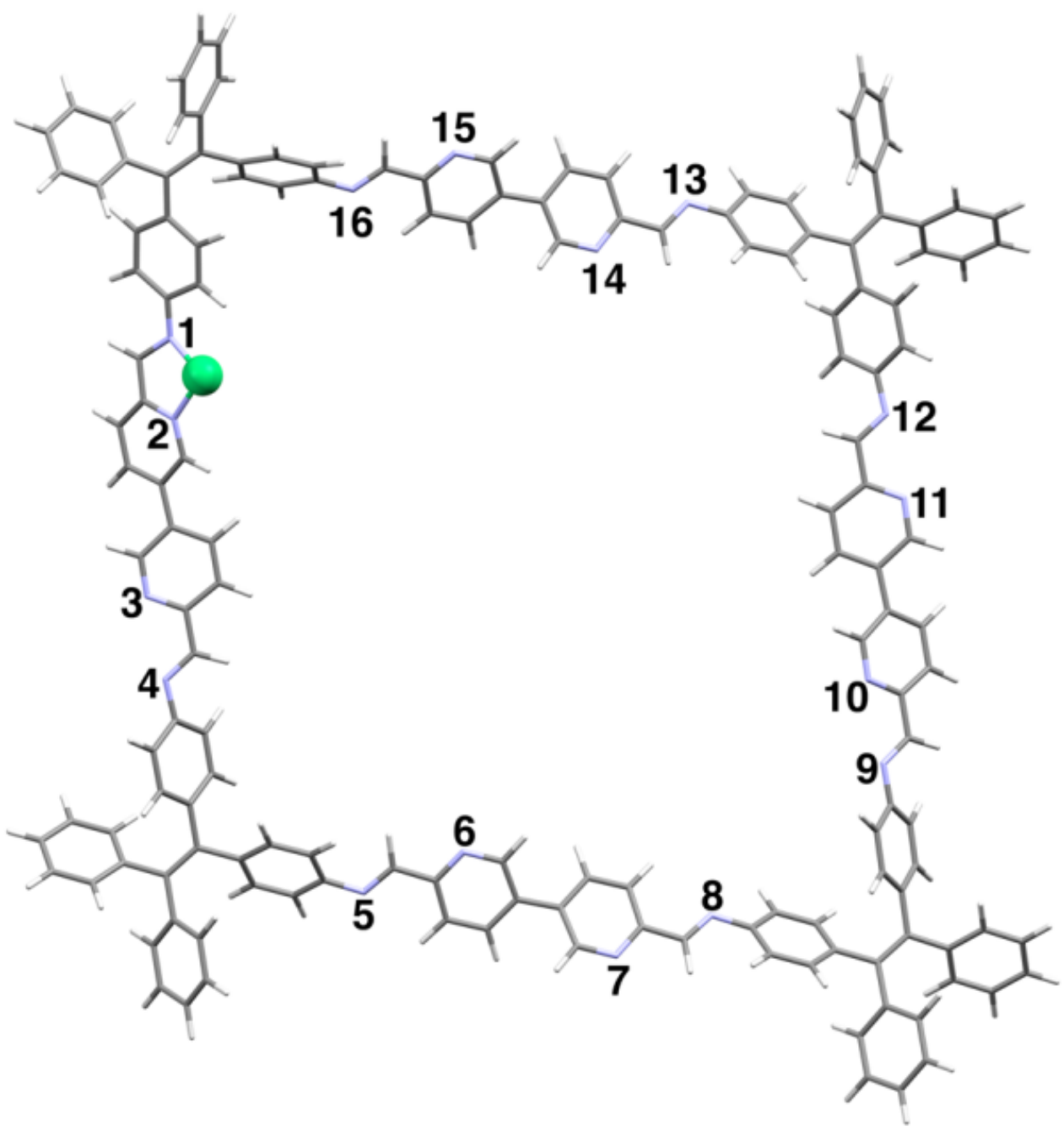

Fig S16: Structural representation of the Ni-doped COF illustrating labelled potential hydrogen adsorption sites. The active sites consist of the central Ni atom, and the nitrogen

atoms labelled 1-16. These labelled atoms were selected as candidate adsorption sites for HER investigations and correspond to the atoms analysed (N-1 to N-16) in the charge redistribution heat map.

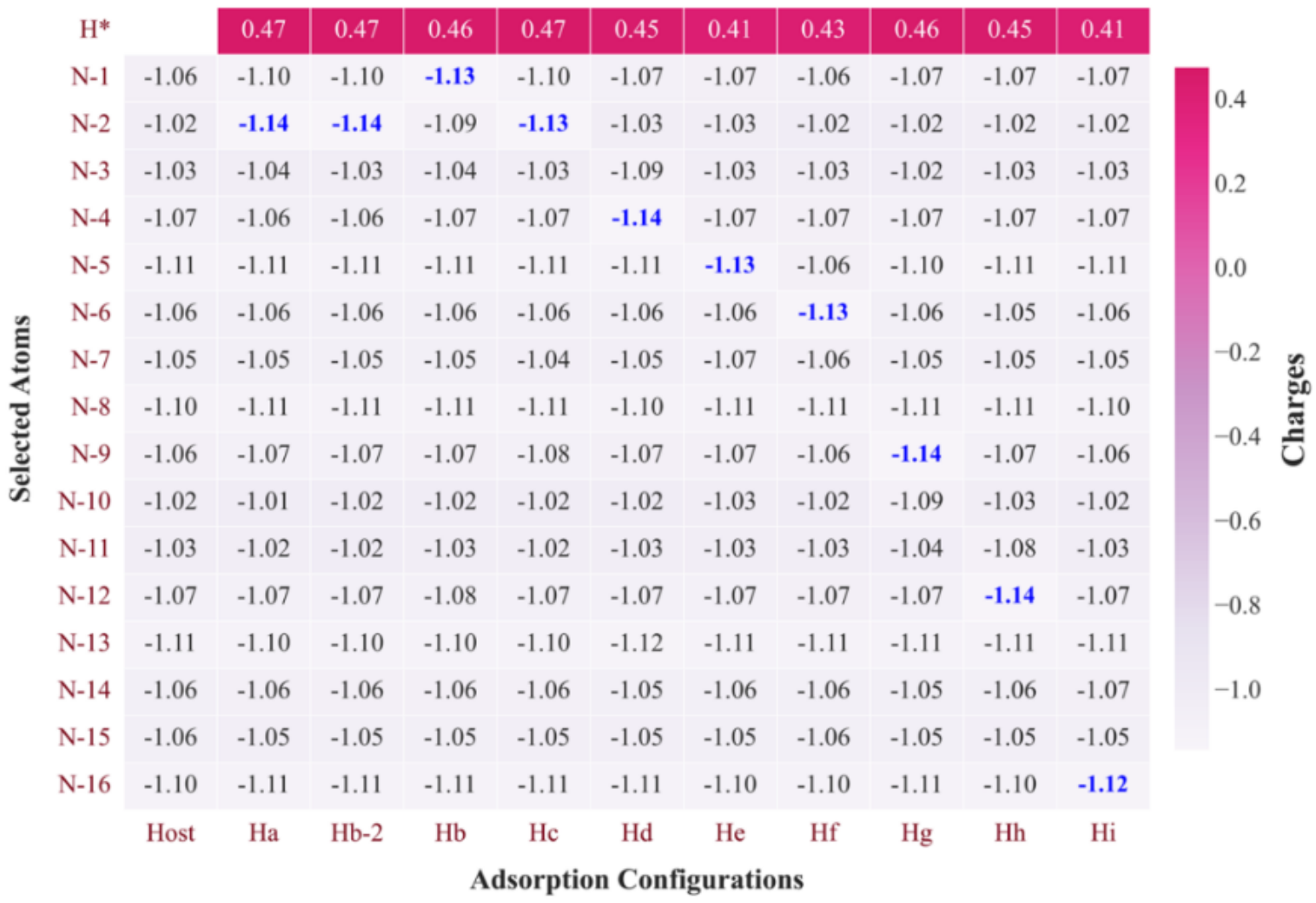

Fig S17: Charge redistribution map obtained from AIM/Bader charge analysis for the COF for different hydrogen adsorption configurations. Columnsrepresentthepristine host and hydrogen adsorption configurations (Ha-Hi), whereas rows correspond to the selected active sites (N-1 to N-16) and the adsorbed hydrogen (H*). The blue values denote the adsorption site directly bonded to H* in each configuration.

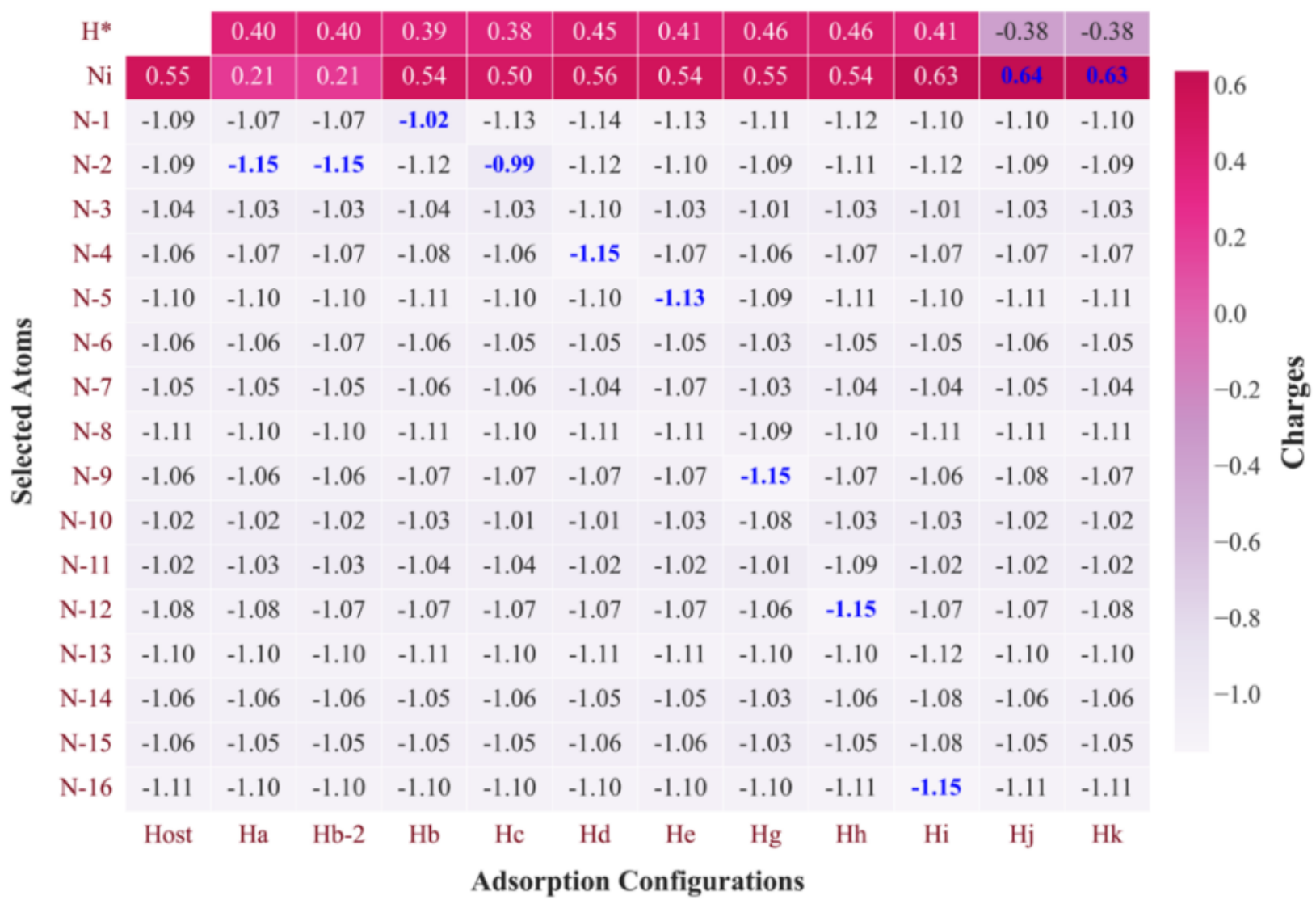

Fig S18: Charge redistribution map obtained from AIM/Bader charge analysis for the Ni-doped COF for different hydrogen adsorption configurations. Columns represent the pristine host and hydrogen adsorption configurations (Ha-Hi), whereas rows correspond to the selected active sites (Ni, and N-1 to N-16) and the adsorbed hydrogen (H^*). The blue values denote the adsorption site directly bonded to H^* in each configuration.

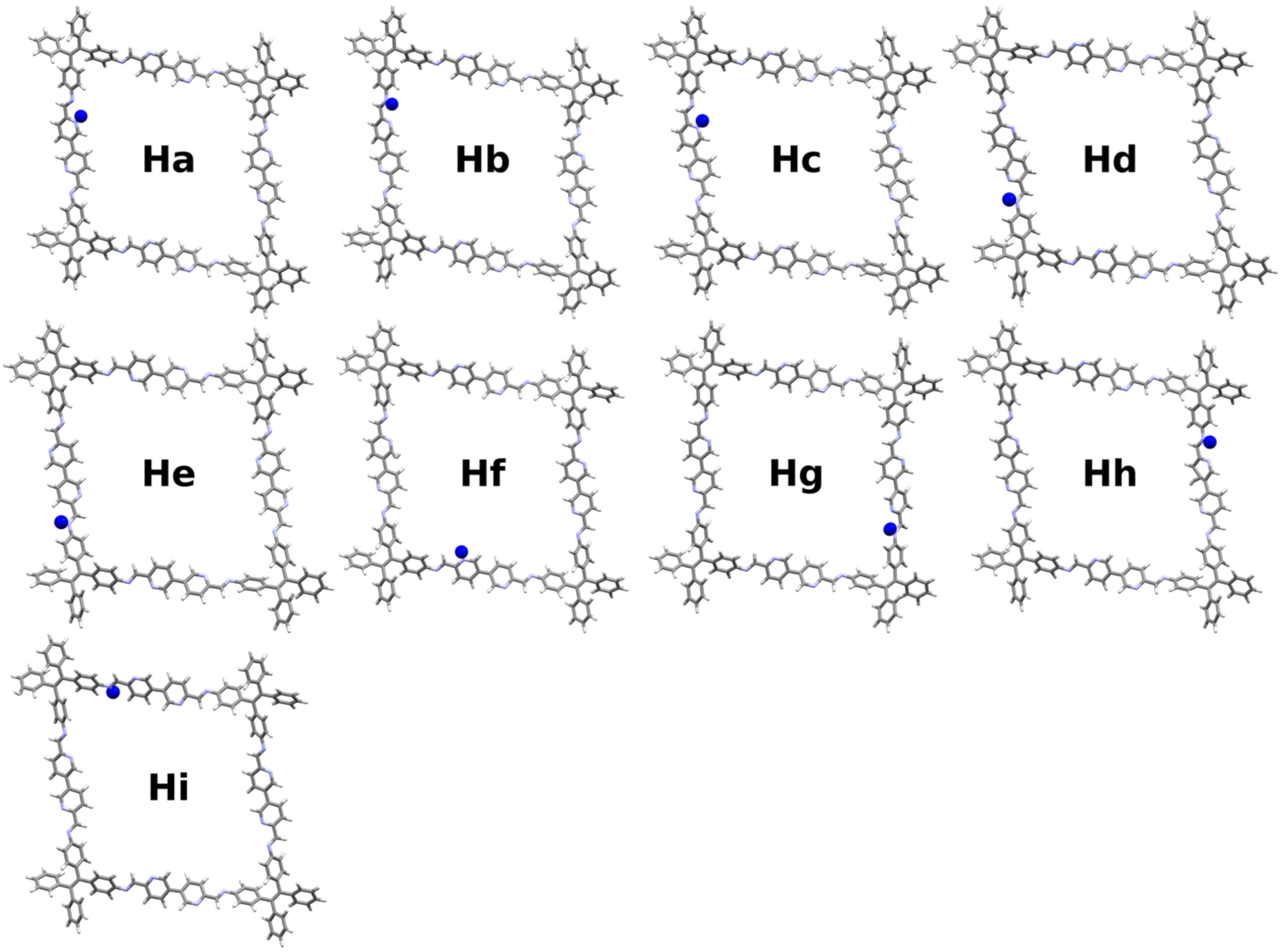

Fig S19: Configurations for the pristine ETTA-BPy-COF investigated as potential active sites for the hydrogen evolution reaction (HER). Configurations Ha–Hi correspond to different hydrogen adsorption positions on the framework nitrogen sites. The adsorbed hydrogen atom (H^*) is represented by the blue sphere.

Table S4: Free Energy of the Absorbed State (ΔG_{H^*}), Chemisorption Energy for the Hydrogen (ΔE_{H^*}), and Zero Point Energy (Δ$EZPE_{H^*}$) for ETTA-Bpy-COF.

Complex	ΔG_{H^*}(eV)	ΔE_{H^*}(eV)	Δ$EZPE_{H^*}$(eV)

Hg	-0.281	-0.373	0.183
Hb	-0.28	-0.373	0.184
Hd	-0.274	-0.364	0.182
Hh	-0.273	-0.365	0.182
Ha	0.012	-0.077	0.168
Hb-2	0.013	-0.077	0.168
Hc	0.016	-0.077	0.169
Hi	0.108	0.031	0.165
He	0.121	0.046	0.164
Hf	0.475	0.393	0.151

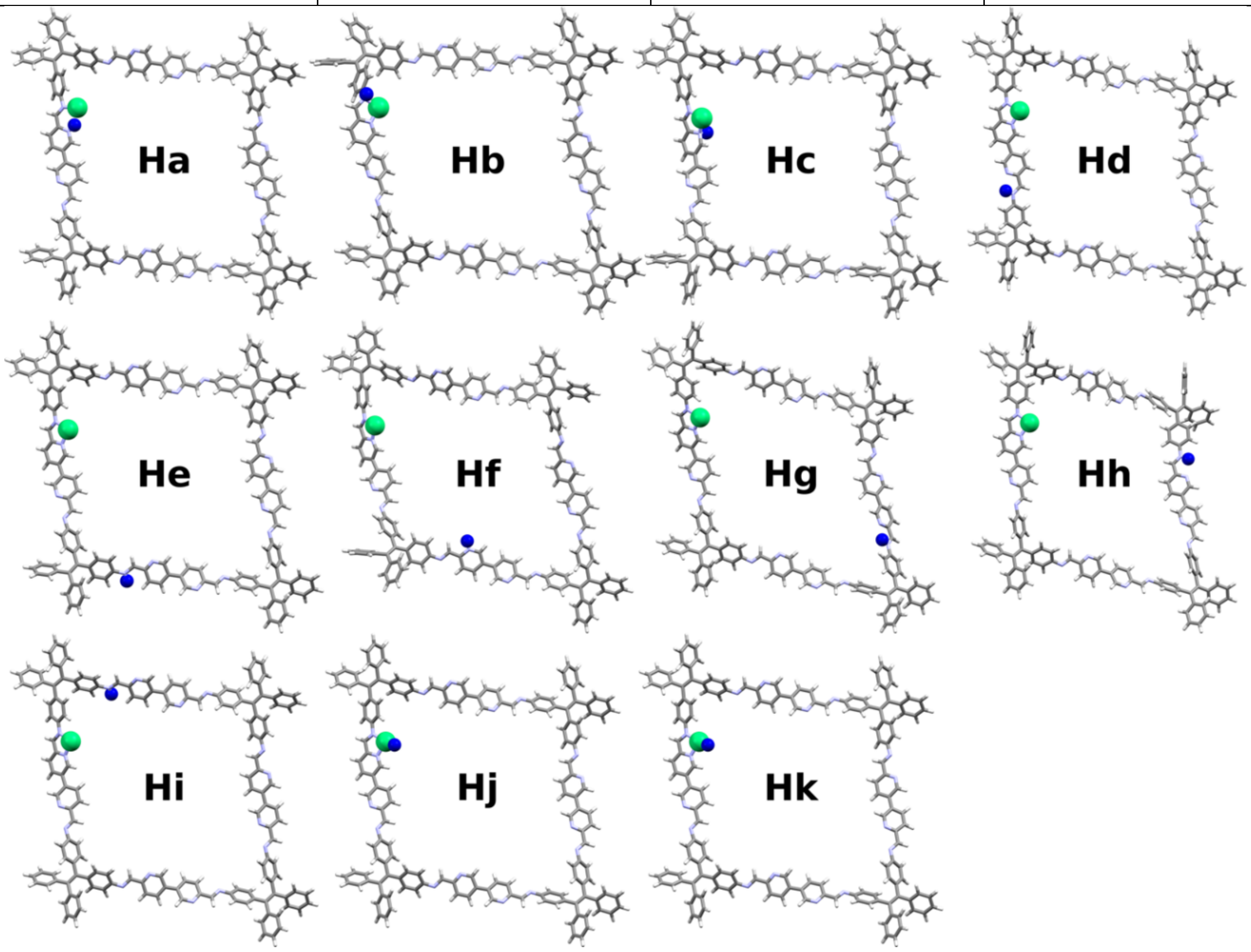

Fig S20: Configurations for the Ni-doped ETTA-BPy COF considered for hydrogen evolution reaction investigations. Configurations Ha-Hk illustrate hydrogen adsorption at different chemically accessible sites in the vicinity of the coordinated Ni centre and

framework nitrogen atoms. The adsorbed hydrogen atom (H*) is shown as a blue sphere, while the doped nickel atom is represented by the green sphere.

Table S5: Free Energy of the Absorbed State (ΔG_{H^*}), Chemisorption Energy for the Hydrogen (ΔE_{H^*}), and Zero-Point Energy (Δ$EZPE_{H^*}$) for ETTA-Bpy- Ni COF.

Complex	ΔG_{H^*}(eV)	ΔE_{H^*}(eV)	Δ$EZPE_{H^*}$(eV)
**Hj**	-0.971	-1.134	0.06
**Hk**	-0.97	-1.134	0.061
**Hd**	-0.957	-1.127	0.196
**Hh**	-0.858	-1.028	0.18
**Hi**	-0.474	-0.641	0.164
**He**	-0.442	-0.612	0.166
**Hb**	-0.003	-0.236	0.193
**Hc**	0.552	0.241	0.173
**Ha**	0.785	0.558	0.15
**Hb-2**	0.785	0.558	0.15

Section S5:

Table S6(A): A Comparison table for the HER activity of **ETTA-Bpy-COF** and **ETTA-Bpy-Ni COF** with previously reported materials:

Material	Overpotential @ 10 $mAcm^{-2}$(mV)	Tafel slope (mV/decade)	Exchange current density (i_o)	Charge transfer resistance(Ω)	Conditions	Reference
Pt electrode	58	30	2.7 × 10^{-3} A cm^{-2}	2	0.5 M H_2SO_4	18-20

THTNi 2DSP	333	80.5	6×10^{-4} mA cm^{-2}		0.5 M H_2SO_4	21
Cu-CMP850	350	135			1 M KOH	22
TpPAM	250	106	2.4×10^{-4} A cm^{-2}	90.34	0.5 M H_2SO_4	23
Ru@COF	212	79		138	1.5 M H_2SO_4	24
SB-PORPy COF	380 @ 5 mA.cm-2	116	5 mA cm^{-2}	145	0.5 M H_2SO_4	25
CoP nanosheets	100	76	0.67 mA cm^{-2}	20	1.0 M KOH	26
MoCx	142	53	0.023 mA cm^{-2}		0.5 M H_2SO_4	27
Mo2C@NPC	260	126.4	1.9×10^{-3} A cm^{-2}		0.5 M H_2SO_4	28
C-Fe, Co-COF	260	150	6.1 mA cm^{-2}		0.1 M KOH	29
TFPB-PAM	185	68.5	4.9×10^{-4} A cm^{-2}	80	0.5 M H_2SO_4	30
NENU-500	237	96	0.139 mA cm^{-2}	28	0.5 M H_2SO_4	31
PETA-PAMCOF	261	122	3.7×10^{-4} A cm^{-2}	62.22	0.5 M H_2SO_4	32
TAT-TFPB	414	100		3.97	0.5M H_2SO_4	33
TAT-TFBE	222	33.7		3.01	0.5M H_2SO_4	33
TAT-TFPEPE	300	73		3.76	0.5M H_2SO_4	33

PCTF-5500	456	650		490	0.5M H_2SO_4	34
PCTF-10400	244	412		35.03	0.5M H_2SO_4	34
PCTF-10500	227	342		11.6	0.5M H_2SO_4	34
PCTF-30500	255	601		390.3	0.5M H_2SO_4	34
Co-MOF	237	117		49.7	1M KOH	35
aCo-MOF	148	110.3		7.71	1M KOH	35
Ni@COP	290	112		5.89	0.5 M H_2SO_4	36
**ETTA-BPy-COF**	**247**	**88**	**$2.1x10^{-4}$ A cm^{-2}**	**70.3**	**0.5M H_2SO_4**	**This work**
**ETTA-BPY-Ni COF**	**214**	**61**	**2.78 x 10^{-4} A cm^{-2}**	**48.4**	**0.5MH_2SO_4**	**This work**

Table S6(B): A Comparison table for the HER activity of **ETTA-Bpy-COF** and **ETTA-Bpy-Ni-COF** with previously reported non noble metal catalyst:

Catalyst	Electrolyte	η_{10} (mV) @ 10 mA cm^{-2}	Tafel slope (mV dec^{-1})	Reference
Ni-Mo Nano-powder	1M KOH	78	35	*ACS Catal.* 2013, **3**, 166–169
$Fe_{0.5}Co_{0.5}P$/CC nanowire array	0.5 M H_2SO_4	37	30	*Nano Lett.* 2016, **16**, 6617–6621
Ni_2P NS/CC	0.5 M H_2SO_4	75	51	*Nanoscale* 2014, **6**, 13440–13445

MoS_2/RGO hybrid	0.5 M H_2SO_4	150	41	*J. Am. Chem. Soc.*2011, **133**, 7296–7299
FeP NAs/CC	0.5 M H_2SO_4	58	45	*ACS Catal.*2014, **4**, 4065–4069
FeP_2 Nanowire	0.5 M H_2SO_4	61	37	*Chem. Commun.*2016, **52**, 2819–2822.
mp-Ni2P/Ni foam	0.5 M H_2SO_4	140	71.6	*ACS Appl. Mater. Interfaces* 2017, **9**, 38, 32812–32819
FeP/GS	0.5 M H_2SO_4	123	50	*Chem. Commun.*2014, **50**, 11554–11557
WP/Ti	0.5 M H_2SO_4	120	54	*Chem. Commun.*2014, **50**, 11026–11028.

Section S6:

References:

1.F. Neese, Approximate second-order SCF convergence for spin unrestricted wave functions, *Chem. Phys. Lett.*, 2000, **325**, 93–98.

2. F. Neese, Software update: The ORCA program system-Version 5.0, *WIREs Comput. Mol. Sci.*, 2022, **12**, e1606.

3. F. Neese, F. Wennmohs, U. Becker and C. Riplinger, The ORCA quantum chemistry program package, *J. Chem. Phys.*, 2020, **152**, 224108.

4. S. Grimme, A. Hansen, S. Ehlert and J. Mewes, r2SCAN-3c: A "Swiss army knife" composite electronic-structure method, *J. Chem. Phys.*, 2021, **154**, 064103.

5. S. Lehtola, C. Steigemann, M. Oliveira and M. Marques, Recent developments in Libxc-A comprehensive library of functionals for density functional theory, *SoftwareX*, 2018, **7**, 1-5.

6. L. Wittmann, I. Gordiy, M. Friede, B. Helmich-Paris, S. Grimme, A. Hansen and M. Bursch, Extension of the D3 and D4 London dispersion corrections to the full actinides series, *Phys. Chem. Chem. Phys.*, 2024, **26**, 21379-21394.

7. E. Caldeweyher, J. Mewes, S. Ehlert and S. Grimme, Extension and evaluation of the D4 London-dispersion model for periodic systems, *Phys. Chem. Chem. Phys.*, 2020, **22**, 8499-8512.

8. E. Caldeweyher, S. Ehlert, A. Hansen, H. Neugebauer, S. Spicher, C. Bannwarth and S. Grimme, A generally applicable atomic-charge dependent London dispersion correction, *J. Chem. Phys.*, 2019, **150**, 154122.

9. E. Caldeweyher, C. Bannwarth and S. Grimme, Extension of the D3 dispersion coefficient model, *J. Chem. Phys.*, 2017, **147**, 034112.

10. F. Neese, The SHARK integral generation and digestion system, *J. Comput. Chem.*, 2022, **43**, 180-189.

11. B. Helmich-Paris, A trust-region augmented Hessian implementation for restricted and unrestricted Hartree-Fock and Kohn-Sham methods, *J. Chem. Phys.*, 2021, **154**, 164104.

12. D. Bykov, T. Petrenko, R. Izsák, S. Kossmann, U. Becker, E. Valeev and F. Neese, Efficient implementation of the analytic second derivatives of Hartree–Fock and hybrid DFT energies: a detailed analysis of different approximations, *Mol. Phys.*, 2015, **113**, 1961-1977.

13. F. Neese, An improvement of the resolution of the identity approximation for the formation of the Coulomb matrix, *J. Com.put. Chem.*, 2003, **24**, 1740-1747.

14. Baerends, E. J.; Aguirre, N. F.; Austin, N. D.; Autschbach, J.; Bickelhaupt, F. M.; Bulo, R.; Cappelli, C.; van Duin, A. C. T.; Egidi, F.; Fonseca Guerra, C.; Förster, A.; Franchini, M.; Goumans, T. P. M.; Heine, T.; Hellström, M.; Jacob, C. R.; Jensen, L.; Krykunov, M.; van Lenthe, E.; Michalak, A.; Mitoraj, M. M.; Neugebauer, J.; Nicu, V. P.; Philipsen, P.; Ramanantoanina, H.; Rüger, R.; Schreckenbach, G.; Stener, M.; Swart, M.; Thijssen, J. M.; Trnka, T.; Visscher, L.; Yakovlev, A.; van Gisbergen, S., *J. Chem. Phys.,* 2025, **162**, 162501.

15. J. W. Furness, A. D. Kaplan, J. Ning, J. P. Perdew and J. Sun, Accurate and numerically efficient r2SCAN meta-generalized gradient approximation, *J. Phys. Chem. Lett.*, 2020, **11**, 8208-8215.

16. J. I. Rodríguez, R. F. W. Bader, P. W. Ayers, C. Michel, A. W. Götz and C. Bo, A high performance grid-based algorithm for computing QTAIM properties, *Chem. Phys. Lett.*, 2009, **472**, 149-152.

17. J. I. Rodríguez, A. M. Köster, P. W. Ayers, A. Santos-Valle, A. Vela and G. Merino, An efficient grid-based scheme to compute QTAIM atomic properties without explicit calculation of zero-flux surfaces, *J. Comput. Chem.*, 2009, **30**, 1082-1092.

18. B. E. Conway and G. Jerkiewicz, Relation of energies and coverages of underpotential and overpotential deposited Hat Pt and other metals to the hydrogen evolution reaction, *Electrochim. Acta*, 2000, **45**, 4075-4083.

19. K. Kunimatsu, T.Senzaki, M. Tsushima and M. Osawa, Infrared study of hydrogen adsorption and evolution on a platinum electrode in acidic solution, *Chem. Phys. Lett.*, 2005, **401**, 451-454.

20. A. Logadottir, G. S. Karlberg, J. Rossmeisl, T. Bligaard, J. Greeley, H. Jónsson and J. K. Nørskov, Trends in the exchange current for hydrogen evolution, *Phys. Chem. Chem. Phys.*, 2007, **9**, 3241-3250.

21. R. Dong, M. Pfeffermann, H. Liang, Z. Zheng, X. Zhu, J. Zhang and X. Feng, Large-area, free-standing, two-dimensional conjugated polymer single-layer sheets for high-performance energy storage, *Angew. Chem., Int. Ed.*, 2015, **54**, 12058-12063.

22. S. Cui, M. Qian, X. Liu, Z. Sun and P. Du, Electrocatalytic hydrogen evolution by a cobalt complex immobilized on a graphitic carbon nitride nanosheet, *ChemSusChem*, 2016, **9**, 2365-2373.

23. B. C. Patra, S. Khilari, R. N. Manna, S. Mondal, D. Pradhan, A. Pradhan and A. Bhaumik, A metal-free covalent organic framework as an efficient electrocatalyst for hydrogen evolution reaction, *ACS Catal.*, 2017, **7**, 6120-6127.

24. S. Maiti, A. R. Chowdhury and A. K. Das, Covalent organic framework-based materials for electrocatalytic hydrogen evolution reaction, *ChemNanoMat*, 2020, **6**, 99-106.

25. S. Bhunia, S. K. Das, R. Jana, S. C. Peter, S. Bhattacharya, M. Addicoat, A. Bhaumik and A. Pradhan, A 2D covalent organic framework as an efficient metal-free electrocatalyst for hydrogen evolution reaction, *ACS Appl. Mater. Interfaces*, 2017, **9**, 23843-23851.

26. L. Su, X. Cui, T. He, L. Zeng, H. Tian, Y. Song, K. Qi and B. Y. Xia, Ultrathin two-dimensional covalent organic framework nanosheets as efficient metal-free electrocatalysts for the hydrogen evolution reaction, *Chem. Sci.*, 2019, **10**, 2019-2024.

27. H. B. Wu, B. Y. Xia, L. Yu, X.-Y. Yu and X. W. Lou, Porous molybdenum carbide nano-octahedrons synthesized via confined carburization in metal–organic frameworks for efficient hydrogen production, *Nat. Commun.*, 2015, **6**, 6512.

28. J.-S. Li, Y. Wang, C.-H. Liu, S.-L. Li, Y.-G. Wang, L.-Z. Dong, Z.-H. Dai, Y.-F. Li and Y.-Q. Lan, Coupled molybdenum carbide and reduced graphene oxide electrocatalysts for efficient hydrogen evolution, *Nat. Commun.*, 2016, **7**, 11204.

29. D. Wu, Q. Xu, J. Qian, X. Li and Y. Sun, A two-dimensional covalent organic framework as an efficient metal-free electrocatalyst for hydrogen evolution, *Chem. Eur. J.*, 2019, **25**, 3105–3111.

30. A. Pradhan and R. N. Manna, Covalent organic framework-derived porous carbon as an efficient electrocatalyst for hydrogen evolution reaction, *ACS Appl. Polym. Mater.*, 2021, **3**, 1376–1384.

31. J.-S. Qin, D.-Y. Du, W. Guan, X.-J. Bo, Y.-F. Li, L.-P. Guo, Z.-M. Su, Y.-Y. Wang, Y.-Q. Lan and H.-C. Zhou, Ultrastable porous coordination frameworks for highly efficient electrocatalytic hydrogen evolution, *J. Am. Chem. Soc.*, 2015, **137**, 7169–7177.

32. S. Samanta, S. Khatun, I. Mukherjee, S. Maity, M. A. Addicoat and A. Pradhan, A metal-free perylene–porphyrin based covalent organic framework for electrocatalytic hydrogen evolution, *Sustain. Energy Fuels*, 2024, **8**, 4183–4191.

33. S. Karak, K. Koner, A. Karmakar, S. Mohata, Y. Nishiyama, N. T. Duong, N. Thomas, T. G. Ajithkumar, M. S. Hossain, S. Bandyopadhyay, S. Kundu and R. Banerjee, Morphology tuning via linker modulation: metal-free covalent organic nanostructures with exceptional chemical stability for electrocatalytic water splitting, *Adv. Mater.*, 2024, **36**, 2209919.

34. H. S. Jena, C. Krishnaraj, B. K. Satpathy, K. S. Rawat, K. Leus, S. K. P. Veerapandian, R. Morent, N. De Geyter, V. Van Speybroeck, D. Pradhan and P. Van Der Voort, Phosphorus covalent triazine framework-based nanomaterials for electrocatalytic hydrogen evolution reaction, *ACS Appl. Nano Mater.*, 2023, **6**, 22684–22692.

35. N. Hussain and S. M. Mobin, Expedited electrochemical activation of cobalt metal–organic frameworks via undercoordination chemistry for robust bifunctional electrocatalytic oxygen evolution reaction and hydrogen evolution reaction, *Chem. Mater.*, 2025, **37**, 8302–8312.

36. A. Samal, D. K. K. Kori, A. Jain and A. K. Das, A nickel-doped two-dimensional covalent organic polymer (2D-COP) for electrocatalytic hydrogen evolution reaction, *ACS Appl. Energy Mater.*, 2024, **7**, 2715–2725.
